# Efficient dispersion modeling in optical multimode fiber

**DOI:** 10.1038/s41377-022-01061-7

**Published:** 2023-02-01

**Authors:** Szu-Yu Lee, Vicente J. Parot, Brett E. Bouma, Martin Villiger

**Affiliations:** 1 Harvard Medical School and Massachusetts General Hospital, Wellman Center for Photomedicine, Boston, MA 02114 USA; 2 Harvard-MIT Health Sciences and Technology, Massachusetts Institute of Technology, Cambridge, MA 02140 USA; 3 Institute for Biological and Medical Engineering, Pontificia Universidad Católica de Chile, Santiago, 7820244 Chile; 4 Institute for Medical Engineering and Science, Massachusetts Institute of Technology, Cambridge, MA 02140 USA

**Keywords:** Fibre optics and optical communications, Imaging and sensing, Biophotonics

## Abstract

Dispersion remains an enduring challenge for the characterization of wavelength-dependent transmission through optical multimode fiber (MMF). Beyond a small spectral correlation width, a change in wavelength elicits a seemingly independent distribution of the transmitted field. Here we report on a parametric dispersion model that describes mode mixing in MMF as an exponential map and extends the concept of principal modes to describe the fiber’s spectrally resolved transmission matrix (TM). We present computational methods to fit the model to measurements at only a few, judiciously selected, discrete wavelengths. We validate the model in various MMF and demonstrate an accurate estimation of the full TM across a broad spectral bandwidth, approaching the bandwidth of the best-performing principal modes, and exceeding the original spectral correlation width by more than two orders of magnitude. The model allows us to conveniently study the spectral behavior of principal modes, and obviates the need for dense spectral measurements, enabling highly efficient reconstruction of the multispectral TM of MMF.

## Abstract (web summary)

A parametric dispersion model that describes mode mixing in multimode fiber enables calibration of the fiber’s multispectral transmission matrix with significantly fewer measurements than existing approaches.

## Graphical abstract

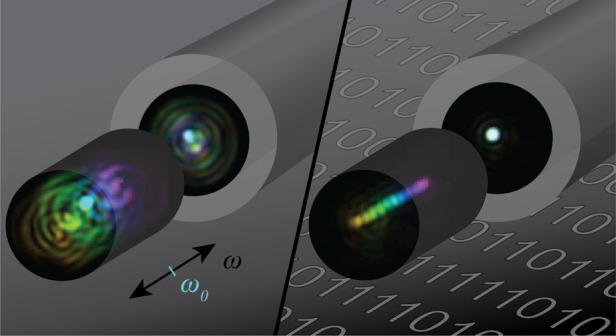

## Introduction

Managing and controlling optical scattering in complex or disordered media has enabled vast new possibilities for imaging, sensing, and manipulation in optical engineering and physics, including communications^1,2^, biomedical optics^3–6^, defense^7^, mesoscopic physics^8^, and quantum optics^9^. Close attention has been paid to calibration methods for compensating the seemingly chaotic transmission through complex media at single wavelengths^3,10,11^. Dispersion, due to geometric effects as well as material properties, inflicts additional spectral scrambling and remains a pervasive and significant technical impediment for multi-color or broadband applications in complex media^3^. Light transport through complex media results in independent intensity distributions beyond a narrow spectral correlation range^12,13^, to the advantage of spectrometry^14,15^, but requiring independent calibration at many frequencies for accurate multispectral wave-control^16–18^. Inconveniently, this results in burdensome measurement time and data storage, which scale linearly with spectral bandwidth and resolution-determined sampling rate^16^.

Optical multimode fiber (MMF) has emerged as an ideal tool for studying transmission through complex media attributed to its high throughput with low loss, defined degrees of freedom, small form factor, controllable geometry, and remarkable dispersion^2,14,19–23^. Principal modes (PMs), the eigenmodes of the group-delay operator, define pairs of specific input and output mode patterns that are unaffected by a change in wavelength^1,24,25^. PMs transmit pulses with a characteristic delay free of temporal scattering into a defined spatial output pattern. Yet, the superposition of PMs that generalizes to an arbitrary input pattern results in a chaotic output that is very sensitive to a change in wavelength. The chromato-axial memory effect has been shown to link a spectral shift of the input illumination with an axial homothetic dilation of the output speckle pattern^26^. Whether this effect is applicable to all available spatial channels stands to be investigated, but attests to a highly deterministic wavelength-dependence of the MMF transmission matrix (TM). Many practical applications require accurate spatio-spectral control and knowledge of the full, spectrally resolved TM. Improved understanding of dispersion and mode mixing in MMF remains an imperative step towards efficient calibration of multispectral TMs (msTMs) and enabling applications associated with multispectral and broadband light transport through MMF.

**Fig. 1:**
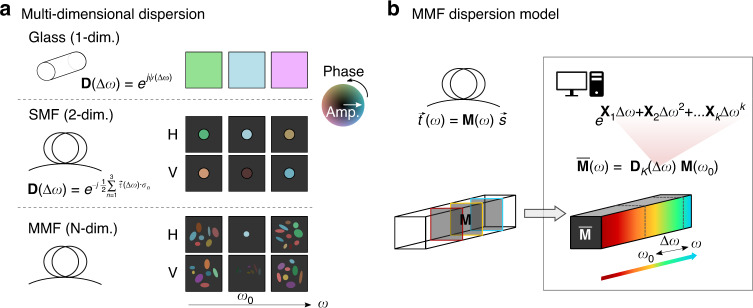
Concept of parametric dispersion modeling. **a** For wave propagation through an isotropic medium such as glass, dispersion due to frequency difference $\Delta \omega$ amounts to a scalar phase term, *ψ*. In single mode fiber (SMF), the polarization-dependence of residual waveguide anisotropy leads to wavelength-dependent polarization states and polarization mode dispersion (PMD), where $\vec \tau$ is the PMD vector, and the ${{{\mathbf{\sigma }}}}_n$ are the Pauli spin matrices^27^. In MMF, a change in wavelength impacts both the polarization and the spatial modes. **b** All these manifestations of dispersion can be modeled by an exponential of a polynomial in the frequency difference $\Delta \omega$. Specifically, we measure the MMF TM at several discrete optical frequencies and fit these measurements to the corresponding dispersion model with matrix series ${{{\mathbf{X}}}}_k$, referenced at $\omega _0$. We then reconstruct the TM ${{{\bar{\mathbf M}}}}\left( \omega \right)$ at continuous *ω* to predict the full spatio-spectral TM

Here we establish a parametric dispersion model of the optical transmission through MMF. It develops the difference between the TMs at two frequencies as an exponential map, polynomial in the frequency offset. This is inspired by the well-known polynomial scalar phase terms of material dispersion. Furthermore, in single mode fiber, polarization-dependent dispersion is modeled with Jones matrices, described by the exponential map of the special unitary group SU(2), and used to analyze the principal states of polarization mode dispersion^27^. As illustrated in Fig. 1, we extend this concept to the higher algebraic dimension of transmission matrices. Owing to the model’s constrained parameter space, we can fit it to experimental TM measurements at few discrete frequencies and predict the TM over a wide frequency range. We verify the model’s performance experimentally in various types of MMF by comparing the predicted TMs with independently measured TMs. For illustration, we use the predicted TMs to computationally focus through the independently measured TMs and use the higher-order model to investigate the frequency-dependence of its PMs. We discuss the spectral sampling conditions for the discrete frequency measurements and investigate trade-offs between the number of measurements and TM fidelity as well as between the closed-form reconstruction of the linear model and the optimization-based fitting of the higher-order model.

## Results

### Efficient dispersion modeling in MMF with exponential mapping

Coherent monochromatic light transmission through a general medium, including MMF, from an input surface to an output surface in the far field can be described by a complex-valued TM^28^. The TM specifies the linear relationship between pairs of input and output spatial channels sampled at discrete locations on the input and output surfaces, respectively. The TM is generally wavelength dependent

1 $$\vec t\left( \omega \right) = {{{\mathbf{M}}}}\left( \omega \right)\vec s$$

where $\vec t$ and $\vec s$ are the vectorized representations of the output and input fields, respectively, and ${{{\mathbf{M}}}}(\omega )$ is the TM at an optical frequency *ω*. To investigate the dispersion captured by the TM we use an input field $\vec s$ that is independent of frequency *ω*. The instantaneous dispersion, relating the frequency dependence of the output field to itself, $\frac{{\partial \vec t}}{{\partial \omega }} = {{{\mathbf{m}}}}\vec t$, is described by the differential matrix

2 $${{{\mathbf{m}}}}\left( \omega \right) = \frac{{\partial {{{\mathbf{M}}}}\left( \omega \right)}}{{\partial \omega }}{{{\mathbf{M}}}}^{ - 1}\left( \omega \right)$$

For a unitary TM, the differential matrix is skew-Hermitian, leading to purely imaginary eigenvalues that identify the group delays of individual PMs, defined by the corresponding eigenvectors. The conventional time-delay operator $- j{{{\mathbf{M}}}}^{ - 1}\left( \omega \right)\frac{{\partial {{{\mathbf{M}}}}\left( \omega \right)}}{{\partial \omega }}$^29^ acts on input PMs, which are related to the output PMs through Eq. 1 and by multiplication with –*j* features real-valued eigenvalues.

If we assume that the differential matrix **m** describes a constant time delay independent of the frequency and with fixed eigenvectors, i.e., PMs, we can integrate Eq. 2 as an initial value problem given ${{{\mathbf{M}}}}\left( {\omega _0} \right)$ to find

3 $${{{\mathbf{M}}}}\left( \omega \right) = e^{{{{\mathbf{m}}}}{{\Delta }}\omega }{{{\mathbf{M}}}}\left( {\omega _0} \right)$$

The variation of **M** due to a change in frequency can in this consequential generalization of PMs be simply accounted for by scaling **m** with the frequency offset Δ*ω*, to adjust the eigenvalues and, hence, the phase delays experienced by PMs at other frequencies. However, this first order description is inherently limited. Not only are the theoretical eigenmodes of a cylindrical waveguide frequency dependent, but fiber bending, twisting, and variations in the fiber geometry all lead to a variation of **m**(*ω*) with frequency. In Supplementary File Sec. 1 we show with a qualitative analysis how transmission through a sequence of MMF segments with disparate but constant **m** generates this frequency dependence.

Analytical integration of Eq. 2 for a general frequency-dependent ${{{\mathbf{m}}}}\left( \omega \right)$ is only possible if ${{{\mathbf{m}}}}\left( {\omega _1} \right)$ commutes with ${{{\mathbf{m}}}}\left( {\omega _2} \right)$ for all *ω*^30^. Instead, we directly develop **M** as the product of ${{{\mathbf{M}}}}\left( {\omega _0} \right)$ at the reference frequency and the exponential map of a matrix Lie algebra **X**

4 $${{{\mathbf{M}}}}\left( {\omega = \omega _0 + {{\Delta }}\omega } \right)\begin{array}{*{20}{c}} { = e^{{{{\mathbf{X}}}}\left( {\omega ,\omega _0} \right)}{{{\mathbf{M}}}}\left( {\omega _0} \right)} \\ { \equiv {{{\mathbf{D}}}}\left( {{{\Delta }}\omega } \right){{{\mathbf{M}}}}\left( {\omega _0} \right)} \end{array}$$

where ${{{\mathbf{D}}}}({{\Delta }}\omega )$ denotes the dispersion matrix, modeling the modification of **M** due to spectral perturbation Δ*ω* referenced to $\omega _0$. We then construct **X** as a series expansion

5 $${{{\mathbf{X}}}}\left( {\omega ,\omega _0} \right) = \mathop {\sum }\limits_{k = 1}^\infty {{{\mathbf{X}}}}_k{{\Delta }}\omega ^k$$

where the complex-valued constant matrix ${{{\mathbf{X}}}}_k$ records the $k^{{{{\mathrm{th}}}}}$ order dispersion. It is important to note that in general there is no closed-form analytical expression relating ${{{\mathbf{X}}}}\left( {\omega ,\omega _0} \right)$ and ${{{\mathbf{m}}}}\left( \omega \right)$. Only when truncating the series at *k* = 1 to obtain the first-order linear model do we find ${{{\mathbf{m}}}} = {{{\mathbf{X}}}}_1$. Summing various orders of ${{{\mathbf{X}}}}_k$ in the exponent is fundamentally different from a sequential product of different order dispersion matrices^31^. Crucially, the exponential map in Eq. 4 linearizes the dispersion matrix and decouples different orders of dispersion into the series of ${{{\mathbf{X}}}}_k$, allowing elegant parameterization of dispersion in a polynomial of ${{\Delta }}\omega$.

### Measurement, fitting, and testing procedure

We measured the polarization-resolved msTM of MMF using a wavelength-tunable laser and an automated measurement system as elaborated in Methods A and illustrated in Supplementary File Fig. S1. Repeating the TM measurement from a starting frequency, $\omega _s$, over a spectral span, Ω, at equidistant optical frequency steps, $\delta \omega$, produces a three-dimensional (3D) msTM, where TMs at ascending frequencies are discretized in $N_\omega$ sampling points, indexed by *n*

6 $${{{\mathbf{M}}}}_n = {{{\mathbf{M}}}}\left( {\omega = \omega _s + n\delta \omega } \right),n = 0,1,2...,N_\omega - 1$$

We then used one or several differently spaced msTMs for fitting our model, referenced at a frequency $\omega _0$ up to *K* orders ${{{\mathbf{D}}}}_K\left( {{{\Delta }}\omega = \omega - \omega _0} \right) = \exp{\left(\mathop {\sum }\nolimits_{k = 1}^K {{{\mathbf{X}}}}_k{{\Delta }}\omega ^k\right)}$. As explained in detail in the following sections, we can derive a first-order model in closed form from a single msTM (Methods B). Since there is no apparent relative loss or gain for transmission through an MMF at different frequencies, we constrain **D** to be a unitary matrix. Fitting higher orders or fitting to multiple, differently sampled msTMs was achieved with gradient descent optimization (Methods C). Under the unitarity constraint, this leads to manifold optimization associated with Riemannian gradient^32,33^, which has emerged as a topic of interest for stabilizing and enhancing the training of deep or recurrent neural networks^34^. An additional strategy fits a linear model to two differently sampled msTMs (Methods D). For fitting, all TMs are projected into a subspace spanned by the leading singular vectors of the TM at $\omega _0$, encompassing the number of modes guided in the fiber, and all TMs are then normalized by their respective Frobenius norms. To estimate the TM at a test frequency $\omega = \omega _0 + {{\Delta }}\omega$, we defined the dispersion compensation ${{{\mathbf{D}}}}_K\left( {{{\Delta }}\omega } \right)$ referenced at $\omega _0$ back in the recording space and computed ${{{\bar{\mathbf M}}}}\left( \omega \right) = {{{\mathbf{D}}}}_K\left( {{{\Delta }}\omega } \right){{{\mathbf{M}}}}\left( {\omega _0} \right)$, where the overline denotes the estimated TM. To evaluate the fidelity of the estimated TM, we computed the cosine similarity, i.e., correlation $C\left( {{{{\bar{\mathbf M}}}}\left( \omega \right),{{{\mathbf{M}}}}\left( \omega \right)} \right)$ between the predicted ${{{\bar{\mathbf M}}}}\left( \omega \right)$ and a separately measured ground truth ${{{\mathbf{M}}}}\left( \omega \right)$ at the same frequency *ω* (Methods E). To visually appreciate the achieved compensation, we used the estimated TMs to computationally focus through the independently measured TMs on a focus location $\vec p$ at varying frequency (see Methods F). This strategy avoids the experimental complication of physically generating the desired wavefronts at distinct wavelengths. We have recently used this approach for computational confocal imaging through MMF without physical wavefront shaping^35^. The focus quality achieved with this approach depends solely on the quality of the TM estimation.

### Ultra-wide hidden spectral correlation

For the initial exploration of the dispersion model, we used a loosely coiled 1m-long 50-μm-core 0.22 numerical aperture (NA) step index (SI) MMF, which supports ~200 modes. The spectral correlation of the fiber’s TM at $\omega _0 = 191$ THz, i.e., the similarity between the TMs at $\omega _0$ and an offset frequency (Methods E), manifests a fast decay with a full width at half maximum (FWHM) of $\delta \nu = 30.43$ GHz (0.26 nm) (see Supplementary File Sec. 4), consistent with previously reported results using MMF with a similar geometry^25^. As a benchmark, we found a similarity of $98.7 \pm 0.14{{{\mathrm{\% }}}}$ between four pairs of repeatedly measured TMs at identical optical frequency. At first glance, the MMF has an independent transmission at a spectral shift beyond $\delta \nu$ and hence low resemblance between different outputs upon just sub-nanometer spectral perturbation. To create a dispersion model, we acquired two msTMs, centered on the reference frequency of $\omega _0 = 191$ THz with $\left( {\omega _s,\delta \omega ,{{\Omega }}} \right) = \left( {190.9,0.01,0.2} \right)$ THz ($N_\omega = 21$) and $\left( {\omega _s,\delta \omega ,{{\Omega }}} \right) = \left( {184,0.467,14} \right)$ THz ($N_\omega = 31$), respectively. The first, narrow msTM served to obtain an initial first order dispersion matrix ${{{\mathbf{D}}}}_1$. Within its narrow spectral range, the first order should be valid and the relative dispersion matrices between adjacent regularly spaced frequencies can be assumed identical, enabling the reconstruction of ${{{\mathbf{D}}}}_1$ in closed form (see Methods B and Supplementary File Sec. 5). $\delta \omega$ for this first msTM was set smaller than half of the original spectral correlation width. This was necessary to ensure that the relative phase between the eigenvalues of ${{{\mathbf{D}}}}_1$ remained smaller than 2*π* without wrapping^36^ to reveal the correct, unambiguous mode-dependent delay (elaborated in Supplementary File Sec. 6). Because experimentally there remained an unknown phase offset between the ${{{\mathbf{M}}}}\left( \omega \right)$ at different frequencies, the global phase of ${{{\mathbf{D}}}}_1$ was set to zero, making these delays relative. The exponential map ${{{\mathbf{X}}}}_{1i}$ was then retrieved by computing the matrix logarithm of ${{{\mathbf{D}}}}_1$, and employed as initial value for fitting a second order dispersion model, using gradient descent to directly optimize the exponential maps ${{{\mathbf{X}}}}_1$ and ${{{\mathbf{X}}}}_2$ (see Methods C and Supplementary File Sec. 5). Conveniently, the formulation of the higher order dispersion optimization does not require regular sampling intervals, and fitting was done simultaneously on both the first narrow msTM and the second, broader msTM spanning 14 THz. The overall time for constructing this $K = 2$ model was $\sim 9$ minutes on an Intel Core CPU (i7-8550U quad-core CPU at 1.8 GHz frequency). Additional higher orders, while taking additional computation time, resulted in negligible improvement on msTM fitting for this fiber.

**Fig. 2:**
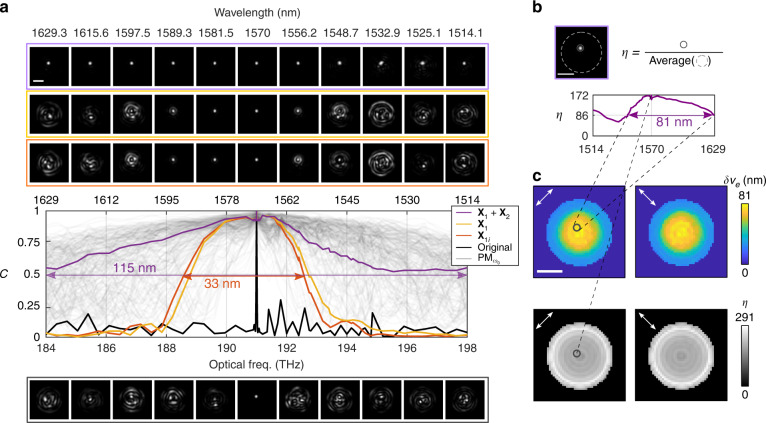
Ultra-wide hidden spectral correlation enables efficient dispersion compensation. **a** The curves correspond to the cosine similarity of independently measured TMs with the reference TM at $\omega _0$ (black), with the first-order estimation using the closed-form reconstruction (orange), and subsequent optimization with *K* = 1 (yellow) or *K* = 2 (purple) using gradient descent optimization, with spectral widths of 0.26, 33, 33, and 115 nm, respectively. Additionally, the correlations of individual PMs computed at $\omega _0$ and applied to all *ω* are shown (gray). The color-coded frames show corresponding synthetic focusing through the MMF at varying wavelengths, where the second order enables excellent focus contrast across the entire spectrum (purple frame). **b** Focus contrast *η* at a spatial channel (gray solid circle) for varying wavelengths using the dispersion model with *K* = 2. The fiber facet is masked from the background (gray dashed circular support). **c** Spatial maps of maximal focus contrast and bandwidth obtained by focusing through all available channels at the fiber core in two polarization states. The scale bars are 20 μm

To evaluate the fidelity of the TM estimation, Fig. 2a plots the cosine similarity $C\left( {{{{\bar{\mathbf M}}}}\left( \omega \right),{{{\mathbf{M}}}}\left( \omega \right)} \right)$ considering ${{{\mathbf{X}}}}_k$ to different orders. To verify the spectral continuity, the frequencies of the ground truth TMs for testing were different from those for fitting and intercalated to the previous grid by half a frequency step. We define the spectral model width, $\delta \nu _e$, as the FWHM bandwidth of the model’s cosine similarity. For the first order dispersion ${{{\mathbf{X}}}}_1$, it is 33 nm, which is $\sim 127$ times broader than the original spectral correlation width $\delta \nu$. The initial estimation captures the linear dispersion in close agreement with computational optimization to the first order. Although the optimized ${{{\mathbf{X}}}}_1$ had a matrix Frobenius norm $489.8$ times larger than ${{{\mathbf{X}}}}_2$, suggesting that only at ${{\Delta }}\omega = 489.8$ THz the two polynomial terms would be equal in Frobenius norm, fitting to second order significantly enhanced the spectral model width, covering the entire 115 nm available from the laser source (purple curve). This is $\sim 442$ times the original $\delta \nu$ and would require almost 1000 TM measurements when directly sampling at $\delta \nu /2$.

To evaluate without using wavefront shaping the effect of the model’s dispersion compensation on the ability to image through a fiber, we used synthetic focusing through the MMF: an input pattern computed to focus through ${{{\bar{\mathbf M}}}}(\omega )$ was numerically propagated through an independently measured ${{{\mathbf{M}}}}(\omega )$ (Methods F). Figure 2a shows a high-quality focus over 33 nm with $K = 1$ (orange and yellow boxes), and across the entire 115 nm spectrum with $K = 2$ (purple box). However, ignoring the model and directly applying the input pattern that focuses at ${{{\bar{\mathbf M}}}}(\omega _0)$ to other wavelengths results in speckle patterns (black box), as expected from the initial poor spectral correlation (black curve). The focus contrast *η*, defined as the peak intensity over the average intensity of all available spatial channels, assesses the spatial dependence of the focus quality. Illustrated in Fig. 2b, the given spatial channel achieves a maximal *η* = 172 at 191 THz and $\eta \,>\, 75$ across the entire spectrum using the *K* = 2 dispersion model. We further evaluated the bandwidth of the focus contrast for all spatial channels by calculating the FWHM of their *η* curves, smoothed with a moving filter of width 5.4 nm. The resulting spatial maps of maximal *η* and bandwidth are shown in Fig. 2c, where the white arrows indicate the polarization states. High focus contrast around 170–200 is maintained over a bandwidth of 70–81 nm at the central core region; towards the cladding the contrast increases to 260–291, accompanied by a reduction in the bandwidth to 22–25 nm. We attributed this variation to the increased transmission loss but higher spatial frequencies of higher order modes that dominate at the core periphery. Dispersion compensation with the fitted model achieves high quality focusing at all available spatial/polarization channels.

Figure 2a also shows the spectral correlations of individual PMs. We computed the input PMs at the center frequency $\omega _0$, applied them to all measured ground-truth ${{{\mathbf{M}}}}\left( \omega \right)$, and then evaluated the cosine similarity of the output PMs at *ω* and $\omega _0$. Most PMs have spectral correlations that exceed the model width, and the first order model is close to the PMs with the narrowest spectral width. The first-order model relies on all PMs to persist with a well-defined phase delay. Once enough PMs exceed their spectral bandwidth, the fidelity of the TMs estimated to first-order degrades. Extending the model to a higher order, in comparison, results in a spectral width approaching the best-performing PMs. To verify the generalizability of our model, we repeated the same experiments with MMF of different types, lengths, coil radii, or from publicly available measurement data sets^37^, and observed reliable fitting with robust performance (see Supplementary File Figs. S7–S9). We found a general reduction in the spectral width of our model with increased fiber length and mode coupling.

### Spectrally variant PMs

The first-order approximation assumes a constant **m** in Eq. 2, independent of the frequency. Even if higher orders ${{{\mathbf{X}}}}_k$ are present, but they commute with each other, i.e., $\left[ {{{{\mathbf{X}}}}_k,{{{\mathbf{X}}}}_{k^\prime }} \right] = 0,k \,\ne\, k^\prime$, then they share the same eigenvectors, and the PMs that they define maintain their profile independent of the wavelength. However, in general, the ${{{\mathbf{X}}}}_k$ series are not commutative and lead to a frequency-dependence of **m** and the PMs. The experimental investigation of this PM dispersion is challenging due to the need for measuring the MMF response and for the noise-sensitive computing of the PMs at many frequencies over a wide spectrum^1^. Conveniently, our MMF dispersion model affords precise numerical characterization of MMF transmission and accurate computation of all spectrally-variant PMs (see Methods G for details).

**Fig. 3:**
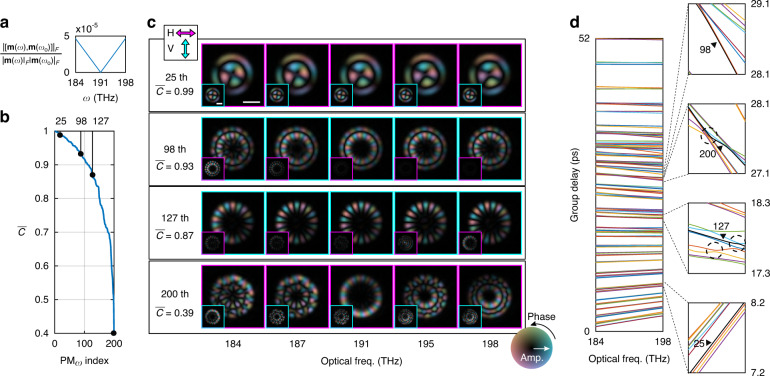
Characteristics of spectrally-variant PMs. **a** Normalized commutativity of ${{{\mathbf{m}}}}\left( \omega \right)$. **b** The permanence of ordered 200 output PMs computed by averaging the spectral correlation across the entire source spectrum. **c** Mode profiles of the $25^{th}$, $98^{th}$, $127^{th}$, and $200^{th}$ output PMs in both *H* and *V* polarization at varying wavelengths and the corresponding permanence. The scale bars are 20 μm. **d** Left: Frequency dependence of individual PM’s group delays in the high order dispersion model; right: zoom insets highlight the group delay evolution of example PMs (black arrows) and degeneracy effects (dashed circles; see main text)

The normalized commutativity between ${{{\mathbf{X}}}}_1$ and ${{{\mathbf{X}}}}_2$ of the second order model presented in Fig. 2 is $\left| {\left[ {{{{\mathbf{X}}}}_1,{{{\mathbf{X}}}}_2} \right]} \right|_F/\left( {\left| {{{{\mathbf{X}}}}_1} \right|_F\left| {{{{\mathbf{X}}}}_2} \right|_F} \right) = 0.0033$. From the model we computed ${{{\mathbf{m}}}}\left( \omega \right)$ following Eq. 2 and plotted the normalized commutativity referenced at $\omega _0 = 191$ THz in Fig. 3a, which increases linearly with frequency offset, indicating at non-commutative **m**. We then studied individual spectrally-dependent PMs and defined their permanence, $\bar C$, as the averaged spectral correlation of a PM across the entire 115 nm spectrum referenced at $\omega _0$. As plotted in Fig. 3b, the permanence of the 200 PMs in this 50-μm-core SI-MMF decreases from 0.99 to 0.40, where the PMs are sorted accordingly. The decreasing permanence clearly reflects the non-commutativity of **m** and hence the limited PM bandwidth. Figure 3c shows several output PMs of different permanence in both *H* (cyan) and *V* (magenta) polarization at varying wavelengths, where the polarization with less energy is plotted in the small insets. Visually, the profiles resemble theoretical Laguerre-Gaussian modes in SI-MMF, which remain constant across wavelength for PMs with high permanence, but change the mode pattern for less permanent PMs. In Fig. 3d, we illustrate the PM dispersion by plotting the spectrally-variant group delays of individual output PMs, relative to the delay of the fundamental mode. The relative group delays span $\sim 52$ ps, consistent with step-index waveguide theory^38^. Interestingly, besides the different nonlinear delays, the PMs exhibit both positive and negative dispersion. In addition, we observed degeneracy effects (dashed circles) between PMs due to unstable eigen-solutions, similar to a previous study^1^. As a result, already the second-order dispersion model enables numerical analysis of spectrally-variant PMs featuring distinctive behaviors. The PM analysis for different MMF can be found in Supplementary File Sec. 7.

### Efficient reconstruction of multispectral TMs

So far, we introduced the high-order dispersion model, demonstrated its ability to estimate MMF transmission well beyond the linear regime, and studied the spectral variation of PMs. Because TMs are prone to perturbation such as fiber bending or temperature change^39,40^, strategies to efficiently characterize spectrally dependent TMs with only few measurements are of high interest for experimental applications. How many monochromatic TM measurements are needed to reliably fit the dispersion model? In the following, we investigated the trade-off between *h*, the number of measurements, and the fidelity of the estimated TM. We did so for both an improved method for efficiently fitting to the first order in a speed-driven approach, and for fitting to the second order to achieve best bandwidth performance. We used the 1m-long 50-μm-core SI-MMF with NA = 0.22 and as in our previous experiments, all results were validated using independently measured ground truth TMs.

#### Speed-driven

**Fig. 4:**
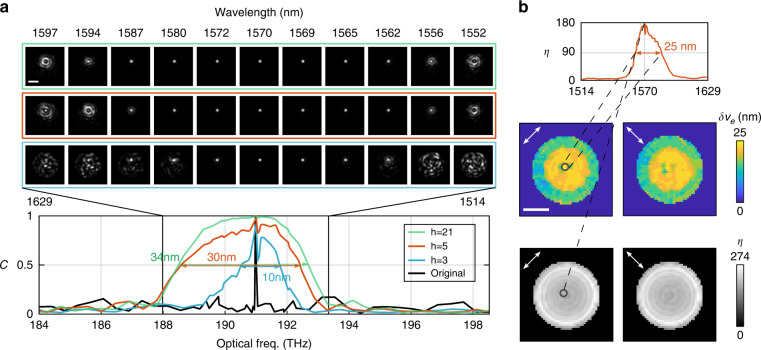
Efficient computation of first order dispersion. **a** Spectral correlation of MMF transmission and focusing through MMF using $h = 3,5,21$ TM measurements. **b** With *h* = 5, we computed the focus contrast *η* at each spatial channel and all wavelengths, and visualized the spatial maps of maximal focus contrast and bandwidth of reconstructed channels for two polarization states. The scale bars are 20 μm

For many applications, speed is of the essence and the bandwidth of the linear regime may be sufficient. As discussed, the dispersion matrix ${{{\mathbf{D}}}}_1$ relating TMs spaced by a fixed frequency offset $\delta \omega$ can be obtained very efficiently in closed form (see Methods B) from at least two TM measurements. Repeated application of ${{{\mathbf{D}}}}_1$ allows extrapolating the TM at discrete frequencies over a wide spectrum but multiplies any error present in the original dispersion matrix, hence favoring a larger $\delta \omega$. Yet, in order to estimate the TM for continuous frequencies, the exponential map ${{{\mathbf{X}}}}_1$ is needed, which can only be recovered unambiguously from ${{{\mathbf{D}}}}_1$ if $\delta \omega \,<\, \delta \nu /2$. To reconcile the need for both large and small $\delta \omega$, we developed a strategy for reconstructing a first order dispersion model in closed form using the first order dispersion matrices of two msTMs having small and large $\delta \omega$, respectively (see Methods D). In short, this method derives the PMs from the dispersion matrix with larger step size and removes the $2\pi$ ambiguity in the phase of its eigenvalues by extrapolating from the correctly resolved fractional phase of the dispersion matrix with smaller $\delta \omega$. The computation for typical msTMs in this case takes 30 seconds on a CPU for TMs with ~200 modes. To investigate the impact of the number of measurements on the model fidelity, we used $N_\omega$ spectral steps for both msTMs, with a total number of $h = 2N_\omega - 1$ TM measurements (subtracted by 1 because of the duplicate TM at $\omega _0 = \omega _s$). We used $\omega _s = 190.9$ THz, $\delta \omega _{small} = 12.2$ GHz and $\delta \omega _{large} = 60.7$ GHz. As plotted in Fig. 4a, the model fidelity using $h = 21$ measurements (green curve) is smooth and has a $\delta \nu _e = 34$ nm. For comparison, the minimal $h = 3$ measurements (blue curve) still achieve a spectral model width of $\delta \nu _e = 10$ nm, with a greatly reduced measurement effort. Using $h = 5$ measurements strikes an optimal balance (orange curve), achieving $\delta \nu _e = 30$ nm, with the best efficiency ($\delta \nu _e/h = 6$ nm) and fast computation time of $\sim 10$ seconds, relaxing the effort by a factor of $\sim 46$ compared to the brute force approach: msTM measurement with half of the original correlation width $\delta \nu /2$ as spectral step across the same 30-nm bandwidth. The images within the blue, orange, and green frames in Fig. 4a visualize the reconstruction accuracy corresponding to different *h* with synthesized focusing through the MMF. For $h = 5$, in Fig. 4b, we exemplify the focus contrast at an individual channel, and plot the spatial maps of the focus contrast and bandwidth. The model covers all available channels with focus contrast and bandwidths varying from 170 to 200 and 17 to 25 nm at the center of the fiber to 250–274 and 15–20 nm at the periphery, respectively.

#### Bandwidth-driven

**Fig. 5:**
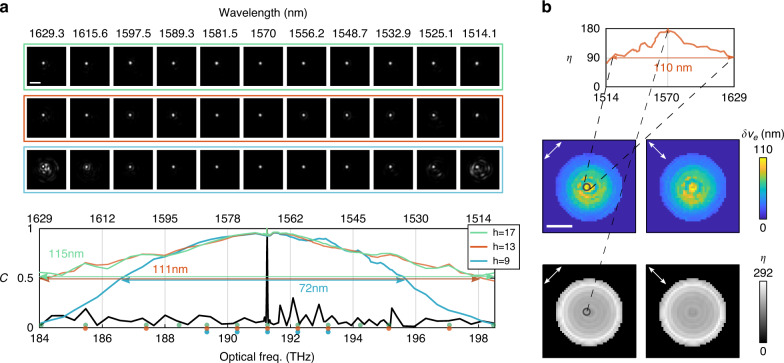
Efficient computation of dispersion up to second order. **a** Spectral correlation of MMF transmission and focusing through MMF using $h = 5 + N_{sub}$ TM measurements. Colored dots indicate the sampled frequencies included in the $N_{sub}$- element subsets, which were used for optimization, in addition to the five measurements of the initial first-order guess. **b** With $h = 13$, we computed the focus contrast *η* at each spatial channel at varying wavelengths, and visualized the spatial maps of maximal focus contrast and bandwidth of reconstructed channels in dual polarization states. The scale bars are 20 μm

For applications where bandwidth is prioritized over speed, but full sampling of the msTM remains impractical, fitting higher order dispersion offers an interesting paradigm to attain broader bandwidth coverage. In an experiment separate from the previous ones, we first constructed a linear dispersion model using five TMs from two msTMs following the aforementioned speed-driven approach, and then selected subsets ($N_{{{{\mathrm{sub}}}}} = 4,8$, or 12) from a third msTM of $\left( {\omega _s,\delta \omega ,{{\Omega }}} \right) = \left( {184,0.933,14} \right)$ THz to optimize dispersion to the second order, fitting to all $h = 5 + N_{{{{\mathrm{sub}}}}}$ available TM measurements. The subsets were not regularly sampled, and the minimal spectral step was determined to avoid phase wrapping issues at higher orders ${{{\mathbf{X}}}}_k$ (see Supplementary File Sec. 6). Figure 5a plots the spectral model fidelity for varying *h* and indicates the corresponding spectral sampling points of the third msTM used during optimization. Here, $h = 13$ ($\delta \nu _e/h = 8.53$ nm) offers the best bandwidth efficiency, being 65.6 times more efficient than the brute force measurement approach across the same bandwidth. Similar to Fig. 4, the focal spot images in the colored frames visualize the focus accuracy corresponding to different *h*. We exemplify the focus contrast and plot the spatial maps of the focus contrast and bandwidth at *h* = 13 in Fig. 5b. The model bandwidth varies from 100–110 nm at the center of the MMF core to 23–28 nm at the periphery, while the contrast increases from 170–190 at the core to 250–292 close to the cladding.

## Discussion

We established an algebraic architecture for modeling modal dispersion and mode coupling in MMF transmission based on exponential mapping. The model not only accommodates PM theory and linear dispersion, but also enables efficient parameterization of higher order dispersion. The achieved wide spectral bandwidth of the modeled MMF transmission demonstrates that spectral MMF transmission through meter-long fibers is highly deterministic and can be measured with high efficiency.

Principal modes have attracted significant attention due to their ability to transmit specific mode patterns independent of a change in wavelength, to first order. However, to obtain full spatio-spectral control, the entire spectrally resolved TM is needed. Our first-order exponential map constructs this TM by accounting for the mode-dependent phase-delays between individual PMs. Yet, while the PMs define a full mode basis, their spectral bandwidth varies substantially^1^. Because the TM depends on all the modes combined, the PMs with the poorest spectral bandwidth limit the bandwidth of the first order model. Interestingly, including higher orders in the exponential map accommodates spectral variation of the PMs and increases the bandwidth of the modeled TM, approaching that of the best-performing individual PMs.

The bandwidth of PMs has been shown to directly depend on the coupling regime within the fiber. With an increase in fiber length, the coupling regime transitions from weakly coupled to strongly coupled when reaching a length identical to the fiber’s transport mean free path, accompanied by a reduction in the PM bandwidth^25^. Our investigation of TM model performance in fibers of different types, lengths, and perturbation indeed observed a decrease in model bandwidth in longer fibers and with increased fiber bending. On the other hand, our model performed well on shorter graded index (GI) MMF, where the almost degenerate modes of higher order mode groups can be considered fully coupled within each group. Model performance was poor, however, on longer GI MMF, accompanied by a collapse of the PM bandwidth. Others have measured significant PM bandwidths in GI MMF as long as 100 m^1^, suggesting that our TM measurements for longer fibers have been compromised, possibly by insufficient phase stabilization.

Our model assumes a unitary dispersion matrix. This constraint was critical to stabilize the gradient descent optimization of higher-order models. Yet, a fundamental limitation of the unitarity constraint, even in weakly coupled fibers, is that the model does not capture the variation in the number of guided modes in MMF as a function of frequency. The rank of the TM should depend on the frequency and cannot be coupled by a unitary matrix between frequencies. Furthermore, even the theoretical mode profiles in a cylindrical waveguide depend on the frequency, putting a similar fundamental limit on PMs. This fundamental limitation may indeed be the limiting factor in the model performance for the loosely coiled SI fibers, as we clearly observe the variation of the number of modes with wavelength (Supplementary File Fig. S3).

Unlike MMF, general complex media are not only fully coupled, but their TMs are also lossy due to backscattering. Moreover, only a subset of all degrees of freedom is available experimentally, and this subset may further vary spectrally. Scattering in complex media likely generates higher dispersion orders, and the limited coverage of degrees of freedom further conflicts with the unitarity constraint. Conceptually, the exponential map could be extended to include attenuation and, hence, define non-unitary dispersion matrices that linearize the relation between the TM of complex media at different wavelengths, but would need improved optimization strategies. Instead of a change in wavelength, Matthès et al.^41^ recently analyzed an operator similar to our dispersion matrix that describes the change in the TM when physically deforming MMF. We speculate that using an exponential map would enable convenient parameterization of this deformation operator.

While our results clearly demonstrate the potential of the dispersion model, our experimental approach of measuring multiple, differently scaled msTMs deserves further exploration. The spectral sampling must allow resolving the modal phase delays, but other sampling schemes could be envisioned. Also, wavelength accuracy, repeatability, and system stability directly influence the fidelity of the TM measurements, in addition to the intrinsic measurement noise. Our empirical investigation of balancing model performance suggests that inclusion of additional wavelengths intrinsically helps to improve the model, because inclusion of TMs repeatedly measured at the same wavelength failed to yield an improvement. Moreover, our measurement methodology compensates for interferometer phase drift during the acquisition of a single TM but results in an unknown phase offset between TMs at different wavelengths. While this is irrelevant to measure the relative mode delays between various modes, it excludes material dispersion from our model. Lastly, we have made little effort to accelerate the steepest gradient descent for high order dispersion estimation. Additionally, the error landscape of Eq. 9 is in general non-convex and the optimization may get caught in a local minimum. This may explain why fitting beyond the second order only yielded modest performance improvement. Techniques such as stochastic gradient descent, momentum, and re-initialization could be helpful in expediting the fitting of higher order dispersion models and avoiding local minima.

Temporal focusing through MMF with arbitrary delays has been demonstrated by exploiting measured msTMs^42^, where MMF with the same geometry as the one used here was fully characterized over a similar spectrum with more than a thousand TM measurements. Alternatively, the TM of scattering media has been measured in the temporal domain, by measuring the interference between the scattered light and a pathlength-controlled reference arm using broadband light^43^. Recently, a rapid multispectral characterization system based on hyperspectral imaging has been introduced for measuring the TM of scattering media^18^, which expedited the data acquisition by nearly 2 orders of magnitude. Our computational dispersion-model-based calibration complements these methods by necessitating only tens of measured TMs when characterizing MMF, which relaxes the hardware complexity of microlens arrays or gratings, brings extra flexibility in system design, and reduces data storage for calibration of the TM over a wide spectrum.

Using the same TM formalism to model free space propagation, the dispersion matrix **D** would be diagonal in the Fourier basis, according to Fresnel diffraction theory, and define a quadratic defocus phase term when reshaping its diagonal into 2D spatial frequencies. Interestingly, for transmission through the 1m-long SI-MMF, reshaping the main diagonal of the recovered **D** into spatial frequency coordinates revealed a pattern similar to a quadratic phase front. This indicates that MMF dispersion is associated with a defocusing effect, which is another observation of the chromato-axial memory effect^26^. Nevertheless, the **D** matrix of the MMF has non-negligible off-diagonal elements, owing to the coupling between spatial modes upon spectral perturbation. While the chromato-axial memory effect has been shown to be valid across a few nanometers in straight MMF of several centimeters in length^26^, our method compensates for additional waveguide dispersion and achieves orders of magnitude broader model bandwidth for all supported modes in loosely coiled, meter-long MMF.

The dispersion model may streamline multispectral characterization of photonic systems in a range of applications. It can be readily applied to, e.g., calibration for MMF-based spectroscopy or nonlinear endoscopy^15,44^. It may spark new multispectral or coherent-based measurement strategies for endomicroscopy through MMF or other multicolor imaging and sensing applications, for dispersion correction in meta-surfaces and materials, and holds promise for both fundamental and applied studies of light transport in MMF. In optical communication systems, MMF features throughput and cost advantages over single-mode fiber, and space-division and wavelength-division multiplexing (SDM and WDM) with MMF have recently been proposed to surpass the Shannon capacity limit of data delivery in single optical fiber^45–47^. The presented model can help to resolve dispersion in the transmission through MMF and may benefit SDM and WDM in the following ways: Combined with wavefront-shaping techniques on the transmitter side, efficient fiber calibration may facilitate physical generation of spectrally-dependent PMs in parallel, or temporal pulse shaping for delivering signals that are favorable to detection; Alternatively, to relax the requirements for active wave control and use only passive multiplexing elements such as photonic lanterns or multiplane light converters on the transmitter side, efficient TM reconstruction at varying wavelengths can be applied on the receiver side and expedite multispectral digital signal processing. As shown in Supplementary Materials, the fiber dispersion model applies well to different GI MMFs, which have even higher number of guided modes than the SI-MMF primarily used in this study, and we expect the model to be suitable for GI-MMF in current SDM applications.

## Conclusion

Our parametric dispersion model efficiently describes transmission through MMF over a wide spectral range. It demonstrates that, far from random, modal crosstalk and dispersion for different wavelengths are highly deterministic and closely related. Use of an exponential map linearizes these relations and affords inter- and extrapolation over continuous wavelengths. The first-order model essentially adjusts for the relative phase of the PMs experienced at different wavelengths. At higher orders, the PMs themselves become wavelength-dependent and extend the bandwidth of the model, approaching the performance of the best individual PMs. The disclosed computational methods open the ability to accurately characterize spectrally resolved TMs from only few spectral measurements, although it is critical that some measurements correctly resolve the mode-dependent delays without ambiguity. Using a second-order model, we accurately estimated the TM of a 1m-long 200-mode SI-MMF over a spectral range exceeding ~442-fold the original spectral correlation width and with 65.6 times higher efficiency than full spectral sampling. The model performed well on various types of MMF and opens new opportunities for fundamental and applied studies in need of accurate spatio-spectral control.

## Materials and methods

### A. Experimental setup

The experimental system is illustrated in Supplementary File Fig. S1. A 1-MHz-line-width wavelength-tunable laser (TSL-510, Santec) with frequency range *ω* = 184–198 THz (1629.3–1514.1 nm in wavelength) and an objective lens (Mitutoyo Plan Apo NIR Infinity Corrected) with a NA of 0.4 were used to generate a 2.5 μm FWHM focus on the input facet of the MMF under test. The focal spot was sequentially coupled into all available input spatial channels, dependent on the fiber geometry, with a two-dimensional (2D) galvanometer mirror scanner (GVSM002, Thorlabs). For every input spatial channel, the laser was switched between horizontal (*H*) and vertical (*V*) linear polarization states using a fiber-based electro-optical phase retarder (PRT1010, Boston Applied Technologies Inc.). We used a InGaAs camera (OW1.7-VS-CL-LP-640, Raptor Photonics) with exposure time of 20 μs and a maximal frame rate of 120 Hz, and employed off-axis digital holography to record the complex-valued fields emitted from the fiber output facet in response to all possible MMF input realizations. The output was projected on the two orthogonal polarization states using a beam displacer (BD40, Thorlabs) for simultaneous, polarization-diverse measurements. Interleaved with the different input realizations in the acquisition of a monochromatic TM, a reference input mode was repeatedly visited for phase tracking and system stabilization. We demodulated the recorded interference patterns by taking into account the spectral variation of the off-axis carrier (see Supplementary File Sec. 3), corrected for phase tracking, flattened output images into column vectors, and constructed a full monochromatic TM at the current operating wavelength (or optical frequency). The overall acquisition time of one monochromatic TM was within several seconds, and was repeated at different wavelengths for the acquisition of msTMs. Since the optical frequency sweep was relatively slow compared to system phase drifting, we could not stabilize the phase offsets between individual TMs.

### B. Estimation of the first order dispersion

For fitting the model, we projected all TMs into the subspace, ${{{\mathbf{U}}}}^{\dagger} {{{\mathbf{MV}}}}$, where **U** and **V** are the first *Q* left and right singular vectors of the TM at $\omega _0$, and *Q* is the number of modes of the MMF. In this subspace the experimental TMs are full rank and invertible. To compute the first order dispersion matrix ${{{\mathbf{D}}}}_1$ from a msTM measured at regular frequency intervals $\delta \omega$, we first aligned the global phase offsets between the TMs at different frequencies by computing the phase between consecutive pairs of complex-valued TMs, corresponding to the phase of the trace of their respective inner product, and correcting the msTM with their cumulative sum. Then we computed ${{{\tilde{\mathbf D}}}}_1\left( {\delta \omega } \right)$, where the tilde indicates a non-unitary ${{{\mathbf{D}}}}_1$, by solving the least squares optimization problem

7 $$\mathop {{{{{\mathrm{argmin}}}}}}\limits_{{{{\tilde{\mathbf D}}}}_1} \mathop {\sum }\limits_{n = 1}^{N - 1} \left\| {{{{\mathbf{M}}}}_{n + 1} - {{{\tilde{\mathbf D}}}}_1{{{\mathbf{M}}}}_n} \right\|_F^2$$

where $\left\| \cdot \right\|_F$ is the matrix Frobenius norm (L2-norm). The solution to this minimization problem can be derived analytically

8 $${{{\tilde{\mathbf D}}}}_1 = \left( {\mathop {\sum }\limits_{n = 1}^{N - 1} {{{\mathbf{M}}}}_{n + 1}{{{\mathbf{M}}}}_n^{\dagger} } \right)\left( {\mathop {\sum }\limits_{n = 1}^{N - 1} {{{\mathbf{M}}}}_n{{{\mathbf{M}}}}_n^{\dagger} } \right)^{{{{\mathrm{ - 1}}}}}$$

Constraining ${{{\tilde{\mathbf D}}}}_1$ to be unitary corresponds to setting the singular values of ${{{\tilde{\mathbf D}}}}_1$ to identity. The overall process is illustrated in Fig. S4a. With ${{{\mathbf{D}}}}_1\left( {\delta \omega } \right)$, we can compute an estimated ${{{\mathbf{X}}}}_{1i} = {{{\mathrm{logm}}}}({{{\mathbf{D}_1}}})/\delta \omega$, which relies on $\delta \omega \,<\, \frac{{\delta \nu }}{2}$ to produce unambiguous delays.

### C. Optimization for higher-order dispersion

We developed a gradient descent optimization algorithm to fit a general dispersion model including higher order terms to msTM measurements without strict condition on the spectral sampling. The formulation in Eq. 7 requires adjusting the unknown phase offset of each measured TM, which becomes difficult beyond the linear dispersion regime. We instead minimized the complementary cosine similarity, $1 - C^2$, similar to ref. ^48^, between each pair of measured ${{{\mathbf{M}}}}\left( \omega \right)$, and estimated $e^{{{{\mathbf{X}}}}\left( {\omega ,\omega _0} \right)}{{{\mathbf{M}}}}\left( {\omega _0} \right)\left( { \equiv {{{\mathbf{D}}}}_K\left( {{{\Delta }}\omega } \right){{{\mathbf{M}}}}\left( {\omega _0} \right)} \right)$ to find the matrices ${{{\mathbf{X}}}}_k$ up to order *K*

9 $$\mathop {{{{{\mathrm{argmin}}}}}}\limits_{{{{\mathbf{X}}}}_k\, \in \,{\frak{g}}} \mathop {\sum }\limits_{n = 1}^N \left( {1 - \left| {tr\left( {{{{\mathbf{M}}}}_n^{\dagger} e^{\mathop {\sum }\limits_{k = 1}^K {{{\mathbf{X}}}}_k{{\Delta }}\omega _n^k}{{{\mathbf{M}}}}\left( {\omega _0} \right)} \right)} \right|^2} \right)$$

where $\frak{g}$ is the group of skew-Hermitian matrices, ${{{\mathbf{M}}}}_n$ is the indexed ${{{\mathbf{M}}}}\left( \omega \right)$ with arbitrary spectral sampling that does not need a regular interval, *tr* indicates the trace of TM products, and we summed over all $N_\omega$ TMs offset from the reference frequency. Note that the skew-Hermitian constraint on ${{{\mathbf{X}}}}_k$ makes ${{{\mathbf{D}}}}_K$ unitary. Taking the absolute squared norm of *C* removes the unknown phase offsets between the TMs. The constraint makes Eq. 9 a manifold optimization problem, which is in general non-convex. Using the estimated first order dispersion ${{{\mathbf{X}}}}_{1i}$ to initialize the first order and initializing the higher ones with zeros, we achieved convergence to a meaningful higher order model by simultaneously optimizing all ${{{\mathbf{X}}}}_k$. For efficient computation, we employed approximated Riemannian gradient descent with an analytical gradient^33^. The overall process is illustrated in Fig. S4b, and details on the gradient descent are available in Supplementary File Sec. 5.

### D. Fast construction of linear dispersion model

Constructing an accurate linear dispersion model without iterative optimization from two msTM measurements with distinct spectral sampling rates, $\delta \omega _{small}$ and $\delta \omega _{large}$, respectively, where $\delta \omega _{small} \,< \,\delta \nu /2$ involves the following steps: First, we compute first order estimations ${{{\tilde{\mathbf D}}}}_1$ independently for both msTMs, using Eq. 7. We then take the eigenvectors of ${{{\tilde{\mathbf D}}}}_1\left( {\delta \omega _{large}} \right)$ from the msTM with larger spectral step as the eigenspace of the improved ${{{\tilde{\mathbf D}}}}_1$. Using these eigenvectors, we diagonalize ${{{\tilde{\mathbf D}}}}_1\left( {\delta \omega _{small}} \right)$ from the msTM with the smaller spectral step, and interpret the phase of the diagonal entries divided by $\omega _{small}$ as the ${{{\mathrm{PM}}}}_{\omega _0}$ delays. Owing to $\delta \omega _{small} \,< \,\delta \nu /2$, these delays are determined without ambiguity, although the small spectral sampling leads to residual errors. Scaling these delays by $\frac{{\delta \omega _{large}}}{{\delta \omega _{small}}}$ should match the phase of the eigenvalues of ${{{\tilde{\mathbf D}}}}_1\left( {\delta \omega _{large}} \right)$, with a 2*π* ambiguity. We can, thus, correct for the residual error present with the small spectral sampling and derive an improved matrix ${{{\tilde{\mathbf D}}}}_1$, and hence the corresponding ${{{\mathbf{X}}}}_{1i}$ . The overall process is illustrated in Fig. S4c.

### E. Calculation of cosine similarity

To quantify the similarity between two TMs, for instance an estimated and a measured TM, or two measured TMs at different frequencies, we computed the cosine similarity between the two corresponding complex-valued matrices, **A** and **B**, as the absolute value of the normalized Frobenius inner product

10 $$C\left( {{{{\mathbf{A}}}},{{{\mathbf{B}}}}} \right) = \frac{{\left| {\mathop {\sum }\nolimits_{ij} a_{ij}^ \ast b_{ij}} \right|}}{{\sqrt {\mathop {\sum }\nolimits_{ij} \left| {a_{ij}} \right|^2} \sqrt {\mathop {\sum }\nolimits_{ij} \left| {b_{ij}} \right|^2} }} = \frac{{\left| tr\left(\mathbf{A}^\dagger\cdot \mathbf{B}\right) \right|}}{{\left\| {{{\mathbf{A}}}} \right\|_F\left\| {{{\mathbf{B}}}} \right\|_F}}$$

where *i* and *j* are the matrix row and column indices, and $a_{ij}$ and $b_{ij}$ are the entries of **A** and **B**, respectively. We then calculated the spectral correlation by comparing a TM at frequency *ω* to the TM at the reference frequency $\omega _0$, $C\left( {{{{\mathbf{M}}}}\left( \omega \right),{{{\mathbf{M}}}}\left( {\omega _0} \right)} \right)$, or the fidelity of a predicted TM by comparing it to its independently measured ground-truth $C\left( {{{{\bar{\mathbf M}}}}\left( \omega \right),{{{\mathbf{M}}}}\left( \omega \right)} \right)$. Alternatively, we used the same expression to compute the spectral correlation of individual PMs, where **A** and **B** are vectors instead of matrices. In all cases, we evaluated the FWHM bandwidth as a measure of spectral width.

### F. Synthesized focusing through MMF

Imaging through MMF can be achieved based on computational reconstruction with synthesized rather than physically generated foci^35,49^. To synthesize focusing through the MMF at *ω*, we right-multiplied the separately measured ground-truth ${{{\mathbf{M}}}}(\omega )$ with the inverse of the modeled transmission ${{{\bar{\mathbf M}}}}^{ - 1}\left( \omega \right)$. This is equivalent to numerically propagating predicted wavefronts through the MMF and focusing on intended output spatial channels. In practice, ${{{\bar{\mathbf M}}}}\left( \omega \right)$ is generally non-square and ill-posed in the recording space coordinates, so we used Tikhonov regularization to approximate its inversion, ${{{\bar{\mathbf M}}}}^{ - 1\left( {tik} \right)}$, with the regularization parameter set to 10% of the largest singular value, as in our previous studies^22,35^. To balance the *H* and *V* polarization detection, we rotated the polarization states of the matrix product by 45°. The spatial basis of the matrix product was then converted from the Fourier domain to real space with inverse Fourier transformation for focus visualization. Reshaping a given column of the matrix product into 2D spatial coordinates reconstructs the image of the corresponding focus.

### G. Calculation of spectrally variant PMs

To obtain the spectrally-variant PMs at a given *ω*, we computed the differential **∆D** with $\delta \omega < \delta \nu /2$

11 $$\Delta {{{\mathbf{D}}}}\left( \omega \right)\begin{array}{*{20}{c}} { = {{{\mathbf{D}}}}\left( {\omega + \delta \omega } \right){{{\mathbf{D}}}}^{ - 1}\left( \omega \right)} \\ { \equiv {{{\bar{\mathbf M}}}}\left( {\omega + \delta \omega } \right){{{\bar{\mathbf M}}}}^{ - 1}\left( \omega \right)} \end{array}$$

This computation was performed directly in the subspace of leading singular vectors, where **D** is full rank and invertible. The eigenvectors of $\Delta {{{\mathbf{D}}}}\left( \omega \right)$ are the output PMs, and the phase of the eigenvalues is associated with the group delays. To compensate for the relative optical path-length difference between the *H* and *V* polarization channels due to the use of the optical beam displacer on the detection pathway (similar to ref. ^1^), we numerically corrected the $\Delta {{{\mathbf{D}}}}\left( \omega \right)$ by applying a spectral linear phase slope to the *V* polarization channel before collecting its eigenvectors and eigenvalues. We repeated the process for varying frequencies *ω*, sorted the PMs by matching them between neighboring *ω*, and calculated the spectral correlation of each PM as the normalized complex vector inner product with itself at $\omega _0$, which is the corresponding permanence. The group delay, $\tau _{pm}$, of an output PM at *ω* is calculated by taking the phase of its corresponding eigenvalue and dividing it by $\delta \omega$. The PMs are then displayed in recording space coordinates, and the fundamental mode is visually identified from its spatial profile to serve as reference for the relative delays of other modes. The PMs with negative delays due to noisy eigenvalues are discarded.

## Data accessibility

Datasets and Matlab code used to generate key results in Figs. 2–4 in the article and Supplementary File Sec. 8 are available in the [Parametric-dispersion-model-for-MMF-transmission] repository, [https://github.com/szuyul/Parametric-dispersion-model-for-MMF-transmission]. Other data that support the findings in the Supplementary File are available from the corresponding author upon reasonable request.

## Supplementary information

Supplementary information
