# Supplementary material for: Efficient dispersion modeling in optical multimode fiber
Source: Light Sci Appl. 2023 Feb 1;12:31. doi: 10.1038/s41377-022-01061-7 (PMC9889807; doi:10.1038/s41377-022-01061-7)
Supplement: Supplementary file 1 — Supplementary information [file 41377_2022_1061_MOESM1_ESM.pdf]

Supplementary Information for
Efficient Dispersion Modeling in Optical Multimode Fiber

Szu-Yu Lee[1,2], Vicente J. Parot[1,3], Brett E. Bouma[1,2,4], and Martin Villiger[1]

[1]Harvard Medical School and Massachusetts General Hospital, Wellman Center for Photomedicine, Boston, Massachusetts 02114, USA
[2]Harvard-MIT Health Sciences and Technology, Massachusetts Institute of Technology, Cambridge, Massachusetts 02139, USA
[3]Institute for Biological and Medical Engineering, Pontificia Universidad Católica de Chile, Santiago 7820244, Chile
[4]Institute for Medical Engineering and Science, Massachusetts Institute of Technology, Cambridge, Massachusetts 02139, USA

Introduction

This supplementary information contains 8 sections. Section 1 presents a mathematical justification of how mode cross-talk results in higher-order dispersion terms in MMF. Section 2 shows the experimental setup of measuring transmission through MMF. Section 3 elaborates on the numerical correction necessary for multi-wavelength off-axis holography. Section 4 shows the DOFs of the MMF based on singular value decomposition and the original spectral correlation. Section 5 describes and illustrates the processing details of modeling dispersion. Section 6 explains the spectral sampling criterion of modeling dispersion. Section 7 shows the generalization of our parametric dispersion model to various MMF and the corresponding performance. Section 8 shows the applicability of our model to different MMF calibration systems using a public data set.

1 Theory

To qualitatively study the dispersion effect in a randomly bent MMF with distributed mode mixing, we modeled the fiber as concatenation of N ideal short segments with individual TMs, $\mathbf{M}_n$, where each of them has wavelength-independent eigenvectors, i.e., propagation-invariant modes, and accordingly linear dispersion. The overall TM of the MMF is then the product of matrix series

$$\mathbf{M} = \mathbf{M}_N \cdots \mathbf{M}_n \cdots \mathbf{M}_3\mathbf{M}_2\mathbf{M}_1 = \overset{\curvearrowright}{\prod}_{i=0}^{N-1}\mathbf{M}_{N-i}, \tag{S1}$$

where the arrow indicates the order of the non-commutative matrix products. Each $\mathbf{M}_n$ has a constant linear dispersion and time delay operator at varying frequency

$$\frac{\partial \mathbf{M}_n(\omega)}{\partial \omega}\mathbf{M}_n^{-1} = \mathbf{m}_n, \tag{S2}$$

such that we can express the TM of each segment as

$$\begin{aligned} \mathbf{M}_n(\omega = \Delta\omega + \omega_0) &= e^{\mathbf{m}_n \Delta\omega}\mathbf{M}_n(\omega_0) \\ &= \sum_{p=0}^{\infty} \frac{(\mathbf{m}_n \Delta\omega)^p}{p!}\mathbf{M}_n(\omega_0), \end{aligned} \tag{S3}$$

where we use series development of the matrix exponential. Since $\mathbf{M}_n$ has wavelength-independent eigenvectors, $\mathbf{m}_n$ and $\mathbf{M}_n(\omega_0)$ share the same set of eigenvectors. We can now express the time delay operator of the overall TM

$$\begin{aligned} \frac{\partial \mathbf{M}(\omega)}{\partial \omega}\mathbf{M}^{-1} &= (\frac{\partial \mathbf{M}_N(\omega)}{\partial \omega}\mathbf{M}_{N-1} \cdots \mathbf{M}_3\mathbf{M}_2\mathbf{M}_1 + \mathbf{M}_N \frac{\partial \mathbf{M}_{N-1}(\omega)}{\partial \omega}\mathbf{M}_{N-2} \cdots \mathbf{M}_3\mathbf{M}_2\mathbf{M}_1 + \cdots \\ &\quad + \mathbf{M}_N\mathbf{M}_{N-1} \cdots \mathbf{M}_2 \frac{\partial \mathbf{M}_1(\omega)}{\partial \omega})\mathbf{M}_1^{-1}\mathbf{M}_2^{-1}\mathbf{M}_3^{-1} \cdots \mathbf{M}_N^{-1} \\ &= \frac{\partial \mathbf{M}_N(\omega)}{\partial \omega}\mathbf{M}_N^{-1} + \mathbf{M}_N \frac{\partial \mathbf{M}_{N-1}(\omega)}{\partial \omega}\mathbf{M}_{N-1}^{-1}\mathbf{M}_N^{-1} + \cdots \\ &\quad + \mathbf{M}_N\mathbf{M}_{N-1} \cdots \frac{\partial \mathbf{M}_1(\omega)}{\partial \omega}\mathbf{M}_1^{-1} \cdots \mathbf{M}_{N-1}^{-1}\mathbf{M}_N^{-1} \\ &= \mathbf{m}_N + \mathbf{M}_N\mathbf{m}_{N-1}\mathbf{M}_N^{-1} + \cdots \\ &\quad + \mathbf{M}_N\mathbf{M}_{N-1} \cdots \mathbf{m}_1 \cdots \mathbf{M}_{N-1}^{-1}\mathbf{M}_N^{-1} \\ &= \mathbf{m}_N + \mathbf{M}_N(\mathbf{M}_N^{-1}\mathbf{m}_{N-1} + [\mathbf{m}_{N-1}, \mathbf{M}_N^{-1}]) + \cdots \\ &\quad + \mathbf{M}_N\mathbf{M}_{N-1} \cdots \mathbf{M}_2(\mathbf{M}_2^{-1}\mathbf{M}_3^{-1} \cdots \mathbf{M}_N^{-1}\mathbf{m}_1 + [\mathbf{m}_1, \mathbf{M}_2^{-1}\mathbf{M}_3^{-1} \cdots \mathbf{M}_N^{-1}]) \\ &= \sum_{n=1}^{N}\mathbf{m}_n + \sum_{k=1}^{N-1}(\overset{\curvearrowright}{\prod}_{i=0}^{k-1}\mathbf{M}_{N-i})[\mathbf{m}_{N-k}, (\overset{\curvearrowright}{\prod}_{j=0}^{k-1}\mathbf{M}_{N-j})^{-1}]. \end{aligned} \tag{S4}$$

We next observe that the first of the serial product terms in Eq. S4 can be expressed using the series development of Eq. S3 as

$$\begin{aligned} \overset{\curvearrowright}{\prod}_{i=0}^{k-1}\mathbf{M}_{N-i} &= \overset{\curvearrowright}{\prod}_{i=0}^{k-1}(\mathbf{I} + \mathbf{m}_{N-i}\Delta\omega + \frac{1}{2}\mathbf{m}_{N-i}^2\Delta\omega^2 + \cdots)\mathbf{M}_{N-i}(\omega_0) \\ &\equiv \sum_{\theta=0}^{\infty}\mathbf{g}_{k,\theta}\Delta\omega^{\theta}, \end{aligned} \tag{S5}$$

which is a polynomial of $\Delta\omega$ with coefficient matrices $\mathbf{g}_{k,\theta}$. Similarly,

$$(\overset{\curvearrowleft}{\prod}_{j=0}^{k-1}\mathbf{M}_{N-j})^{-1} = \overset{\curvearrowleft}{\prod}_{j=0}^{k-1}\mathbf{M}_{N-j}^{-1} = \overset{\curvearrowleft}{\prod}_{j=0}^{k-1}\mathbf{M}_{N-j}^{-1}(\omega_0)(\mathbf{I} - \mathbf{m}_{N-j}\Delta\omega + \frac{1}{2}\mathbf{m}_{N-j}^2\Delta\omega^2 - \cdots) \equiv \sum_{\phi=0}^{\infty}\mathbf{f}_{k,\phi}\Delta\omega^{\phi}. \tag{S6}$$

Plugging Eqs. S5 and S6 in to Eq. S4, we have

$$\begin{aligned}\frac{\partial\mathbf{M}(\omega)}{\partial\omega}\mathbf{M}^{-1} &= \sum_{n=1}^{N}\mathbf{m}_n + \sum_{k=1}^{N-1}\sum_{\theta=0}^{\infty}\mathbf{g}_{k,\theta}\Delta\omega^{\theta}[\mathbf{m}_{N-k}, \sum_{\phi=0}^{\infty}\mathbf{f}_{k,\phi}\Delta\omega^{\phi}] \\ &= \mathbf{s}_0 + \sum_{p=1}^{\infty}\mathbf{s}_p\Delta\omega^p \\ &\equiv \mathbf{m}(\omega).\end{aligned} \tag{S7}$$

As a result, the non-commutativity between $\mathbf{m}_n$, which essentially models the mode cross-talk, leads to the spectral variance in $\mathbf{m}(\omega)$ and hence higher order dispersion upon a $\Delta\omega$ spectral shift. When the $\mathbf{m}_n$ commute with each other, the terms involving commutators in the right-hand side (RHS) in Eq. S7 disappear, leaving the sum over the $\mathbf{m}_n$, and the PMs of the MMF remain wavelength-independent. However, we did not find a compact expression for the solution to Eq. S7.

2 Experimental setup

We developed an automated msTM measurement system illustrated in Fig. S1.

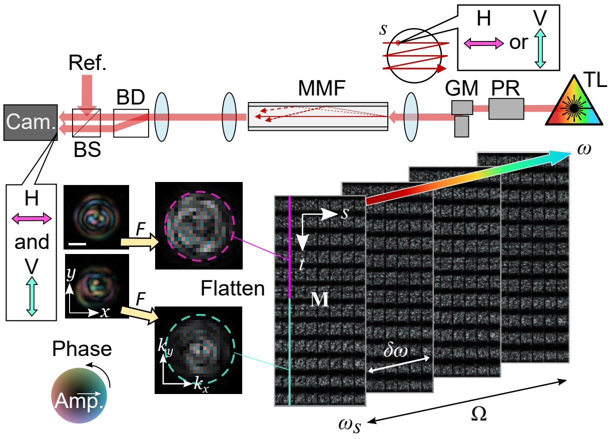

Figure S1: Measurements of the multipsectral transmission through a MMF. The fiber, though drawn as if it were straight, was in fact randomly coiled in experiments. TL: tunable laser. PR: phase retarder. GM: galvanometer scanning mirrors. BD: beam displacer. Ref.: reference wave. Cam.: camera. The focal spot is alternated between H and V by PR and scanned across the MMF input facet by GM, and the output facet is relayed onto the camera, where the two LP states are spatially separated by BD. Although the complex output mode is displayed in real space for better visual appearance, it is stored and analyzed in the Fourier domain. A subset of each TM is shown, where the colored solid vertical lines indicate the vector arranged as an output mode. The color map encodes the complex values, and the scale bar is 20 µm.

3 Correction to spatial channel misalignment

In the off-axis holographic imager setup, the modulation frequency of fringe images is dependent on the effective in-plane momentum component and thus on the operation wavelength [1]. Since we recorded TMs at fixed pixels

in the Fourier domain, the spatial channels were misaligned at different wavelengths. To measure a msTM through identical channels, we need to gauge the varying modulation frequency and co-register the pixels. As shown in Fig. S2(a), we obtained multiple modulated MMF images corresponding to different input realizations at each wavelength, averaged the intensity of the images in the Fourier domain, and isolated the corresponding interference lobes. We repeated the process at decreasing wavelength and tracked the center of the lobes across the laser spectrum. As shown in Fig. S2(b), we then fitted the center trajectory with linear regression, which informs on the required pixel shift in the Fourier domain for a given wavelength. For each TM measured at a frequency ω, we imposed corresponding spatial channel registration, and the center position at varying frequency after the correction is shown in Fig. S2(c). While only correction to H polarized channels is shown, we conducted individual corrections to both polarization channels.

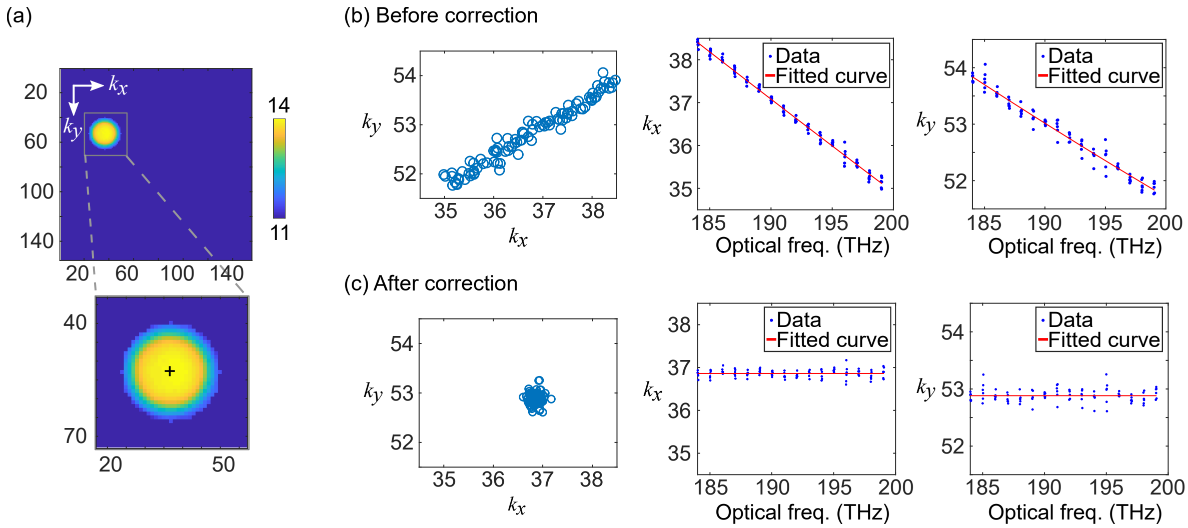

Figure S2: Correction to the varying modulation frequency in multispectral off-axis holography. (a) The intensity of MMF outputs in the Fourier domain in logarithmic scale averaged over several input realizations at $\omega_0 = 191$ THz. We extracted the interference lobe (inset) and tracked the lobe center (black cross) in terms of digitized (k_x, k_y) at varying frequency. (b) Before correction, the center shifts linearly in both k_x and k_y, where we can linearly fit the varying carrier frequency. (c) After correction, the center position only fluctuates due to measurement noise.

4 Characterization of multispectral transmission matrix

The number of guided modes (or DOFs) Q within the MMF can be quantified by performing singular value decomposition (SVD) on each measured TM, counting the singular values (SVs) above a threshold defined as 5% of the largest SV. As shown in Fig. S3(a), for the SI-MMF with 50µm core size and NA 0.22 operated at around 1550 nm, there are $Q\sim200$ DOFs, or populated modes, in each monochromatic TM of ω within the laser tunable range, consistent with the theoretical values from a typical SI-MMF model, and the number of DOFs gradually increases with ω.

We then calculated the spectral correlation of TMs with one at a reference frequency, $C(\mathbf{M}(\omega), \mathbf{M}(\omega_0))$, where $\omega_0 = 191$ THz. As plotted in the solid curves in Fig. S3(b), the original TM spectral correlation without processing manifests a fast decay with a half width at half maximum (HWHM) of $\sim$15.21 GHz (thus the FWHM is $\delta\nu = 30.43$ GHz $= \sim$0.26 nm) due to modal dispersion, consistent with previously reported results with SI-MMFs in weakly scattering regime [2]. As benchmark, we found a $98.7\pm0.14\%$ correlation between two repeatedly measured TMs at an identical optical frequency. The low resemblance between different MMF outputs upon even just sub-nanometer spectral perturbation hence led to the plausible conclusion of independent transmission through MMFs at a spectral shift beyond a coherence length.

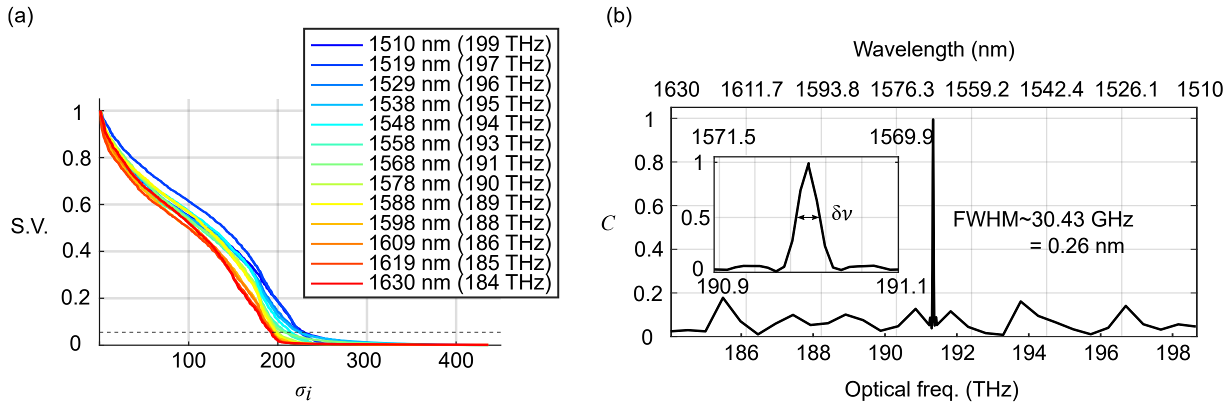

Figure S3: Characteristics of the 1m-long 50-µm-core SI-MMF (a) The number of the modes are ∼200, determined by singular values of TMs and increases with decreasing wavelengths. (b) The spectral correlation of TMs referened at $\omega_0 = 191$ THz. The original $\delta\nu = 0.26$ nm.

5 Data processing framework

After acquiring msTMs, correcting the spatial channel misalignment, and projecting all TMs into the subspace spanned by the singular vectors of the TM at ω_0, we loaded the data into a sequential computational framework to reconstruct the dispersion model. As illustrated in Fig. S4(a), the processing begins by estimating linear dispersion, $\tilde{\mathbf{D}}_1$, with analytical operations from a msTM across a small spectrum, with aligned phase offsets. $\tilde{\mathbf{D}}_1$ is then projected into the subspace of unitary matrices to obtain $\mathbf{D}_1$. $\mathbf{D}_1$ can then be used for either the initialization of higher-order dispersion optimization, or fast construction of an improved linear dispersion model by combining it with a second msTM covering a wider spectrum.

Figure S4(b) shows the pipeline of deriving higher-order dispersion given an estimated $\mathbf{D}_1$. We first computed for $\mathbf{X}_{1i}$ from $\mathbf{D}_1$ as an initial guess of the overall dispersion. To refine $\mathbf{X}_{1i}$ and find high order dispersion $\mathbf{X}_k (k > 1)$ up to K order, we included a msTM across a large spectrum in the fitting set, and minimized the corresponding complementary correlation

$$f(\mathbf{D}_K = e^{\mathbf{X}}) = \sum_{n=0}^{N_\omega} f_n(\mathbf{D}_K) = \sum_{n=0}^{N_\omega} 1 - |tr(\mathbf{M}_n^\dagger \mathbf{D}_K \mathbf{M}_0)|^2. \tag{S8}$$

Here, N_ω is the number of total pairs of matrices $(\mathbf{M}_n, \mathbf{M}_0)$, $\Delta\omega_n$ is the frequency shift between $(\mathbf{M}_n, \mathbf{M}_0)$, tr is the matrix trace, and K is the truncation index of the $\mathbf{X}_k$ series. The challenge is to obtain an analytical gradient of $f(\mathbf{D}_K)$ with respect to the individual orders $\mathbf{X}_k$ of the exponential map $\mathbf{X} = \sum_{k=0}^{K} \mathbf{X}_k \Delta\omega^k$, for efficient steepest gradient descent optimization under the unitary constraint of $\mathbf{D}_K$ (i.e., skew-Hermitian constraint on all $\mathbf{X}_k$). The gradient with respect to $\mathbf{D}_K$ for the complementary correlation of a single pair n is

$$\nabla f_n(\mathbf{D}_K) = -2 \cdot tr(\mathbf{M}_n^\dagger \mathbf{D}_K \mathbf{M}_0)\mathbf{M}_n \mathbf{M}_0^\dagger. \tag{S9}$$

Following Lezcano et al. [3], we can use this gradient to express the derivate with respect to the exponential map $\mathbf{X}$

$$\frac{\partial f_n(e^{\mathbf{X}})}{\partial \mathbf{X}} = e^{\mathbf{X}} (\mathrm{d}\exp)_{-\mathbf{X}} \left(\frac{1}{2}(\nabla f_n(e^{\mathbf{X}})^\dagger e^{\mathbf{X}} - (e^{\mathbf{X}})^\dagger \nabla f_n(e^{\mathbf{X}}))\right). \tag{S10}$$

With respect to a single order of the exponential polynomial, the derivative becomes

$$\frac{\partial f_n(e^{\mathbf{X}})}{\partial \mathbf{X}_k} = \frac{\partial f_n(e^{\mathbf{X}})}{\partial \mathbf{X}} \frac{\partial \mathbf{X}}{\partial \mathbf{X}_k} = \frac{\partial f_n(e^{\mathbf{X}})}{\partial \mathbf{X}} \Delta\omega^k. \tag{S11}$$

Summing over all matrix pairs to obtain the full gradient then gives

$$\frac{\partial f(e^{\mathbf{X}})}{\partial \mathbf{X}_k} = \sum_{n=0}^{N_\omega} \frac{\partial f_n(e^{\mathbf{X}})}{\partial \mathbf{X}} \Delta\omega^k. \tag{S12}$$

The differential of the exponential of matrices $(\mathrm{d}\exp)_{\mathbf{X}}$ can be approximated to machine-precision by computing the exponential of a matrix with twice the width and height of $\mathbf{X}$ [4].

For the gradient descent optimization, we have the iterative update rule

$$\mathbf{X}_k^{i+1} = \mathbf{X}_k^i - \alpha \cdot \frac{\partial f(e^{\mathbf{X}^i})}{\partial \mathbf{X}_k^i}, \tag{S13}$$

where the superscript i is the iteration index and α is the step size. We developed an adaptive update step size for iterations based on backtracking line search with a shrinking/expanding rate of 0.9 [5]. It typically took few hundreds of iterations to reach convergence, and the computation time, depending on the number of matrices and their dimensions (or the DOFs of MMF), ranged from several seconds to tens of minutes. Figure S4(b) shows examples of the spectral perturbation matrix and the first two orders of $\mathbf{X}_k$ series.

Figure S4(c) shows the pipeline of fast construction of linear dispersion model. We computed the eigenvectors of $\tilde{\mathbf{D}}_1(\delta\omega_{large})$ from a msTM with larger step size, and used the eigenvectors to diagonalize and collected the main diagonal of the $\tilde{\mathbf{D}}_1(\delta\omega_{small})$ from a msTM with smaller step size. The eigenvectors and the main diagonal elements allow us to construct a more accurate linear dispersion model $\mathbf{D}_1$ within few seconds.

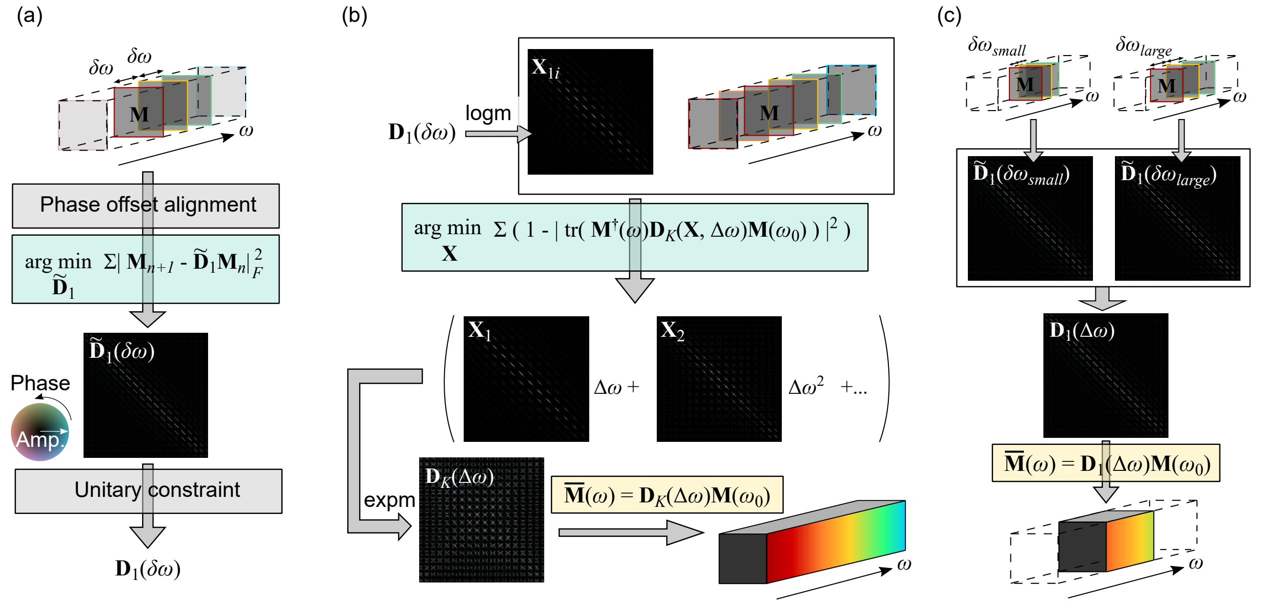

Figure S4: Data processing pipeline for dispersion model construction. (a) We used a msTM with equidistant spectral step size to estimate linear dispersion $\mathbf{D}_1$. (b) We computed $\mathbf{X}_{1i}$ from estimated $\mathbf{D}_1$ and initialized the optimization process for finding higher-order dispersion, $\mathbf{X}_k$, with a msTM across large spectrum. With fitted $\mathbf{X}_k$ series, we can predict the TM at an arbitrary optical frequency. (c) By using two msTMs of different step sizes, we can construct linear dispersion model with high accuracy and time efficiency.

6 Phase wrapping issue

In experiments, we measured TMs at discrete optical frequencies. To construct a dispersion model in Eq. 3 over continuous bandwidth, we need $\mathbf{D}(\omega, \omega_0)$ with correct eigenvalues without 2π phase wrapping ambiguity. With a measured msTM of small enough step size $\delta\omega$ such that the maximal phase difference of eigenvalues of $\tilde{\mathbf{D}}_1(\delta\omega)$ does not exceed 2π, we can derive the correct changing rate of individual eigenvalues (equivalent to the group delays of PMs). In reverse, the temporal spread due to modal dispersion of signals transmitted through MMF imposes the required spectral sampling to correctly resolve all delays.

To show the phase wrapping issue, we constructed two linear dispersion models of the same 1m-long 50-µm-core SI-MMF geometry with two sets of msTM measurements, respectively. The first set has two msTMs of

$(\omega_s, \delta\omega, \Omega) = (190.9612, 0.0122, 0.1216)$ and $(192.3077, 0.3033, 3.033)$ THz, and the second set has two msTMs of $(\omega_s, \delta\omega, \Omega) = (190.4762, 0.0607, 0.6066)$ and $(192.3077, 0.3033, 3.033)$ THz. Note that the two sets of measurements only have difference in the spectral sampling rate of the first msTM, where the sampling rate is respectively above or below the Nyquist criterion, i.e., the minimum spectral sampling rate that avoids aliasing of the relative phase delays in $\tilde{\mathbf{D}}_1$ eigenvalues. Fig. S5(a) shows the eigenvalues of $\tilde{\mathbf{D}}_1(\delta\omega)$ of the first msTM of each set in the complex plane: When above Nyquist sampling rate (top), the angular distribution of the meaningful eigenvalues does not cover the entire plane, and we can retrieve the relative phase offsets of the eigenvalues without ambiguity; However, when below the Nyquist sampling rate (bottom), the eigenvalues spread all over the plane, and we cannot determine the phase offsets due to the 2π ambiguity. We then tested the two models with separately measured TMs at uncalibrated frequencies, which is associated with fractional matrix power of $\tilde{\mathbf{D}}_1(\delta\omega)$ by linearly scaling the eigenvalue phases by a real value. Fig. S5(b) shows the computational spectral correlation after dispersion compensation to the first order. When below the Nyquist sampling rate (bottom), the TM prediction at a frequency off the grid of $\delta\omega$ is inaccurate due to the incorrect phases of eigenvalues. Mathematically, if we develop the fractional matrix power in closed form by diagonalization, since we can add any integer of 2π to the phases of complex eigenvalues without changing the values, finding numerical roots based on commonly used De Moivre's Theorem may lead to a false solution when the Nyquist condition is not satisfied. Generally speaking, the Nyquist rate increases with MMF length and NA, and the required narrower spectral resolution also has implications for the coherence length of the laser source.

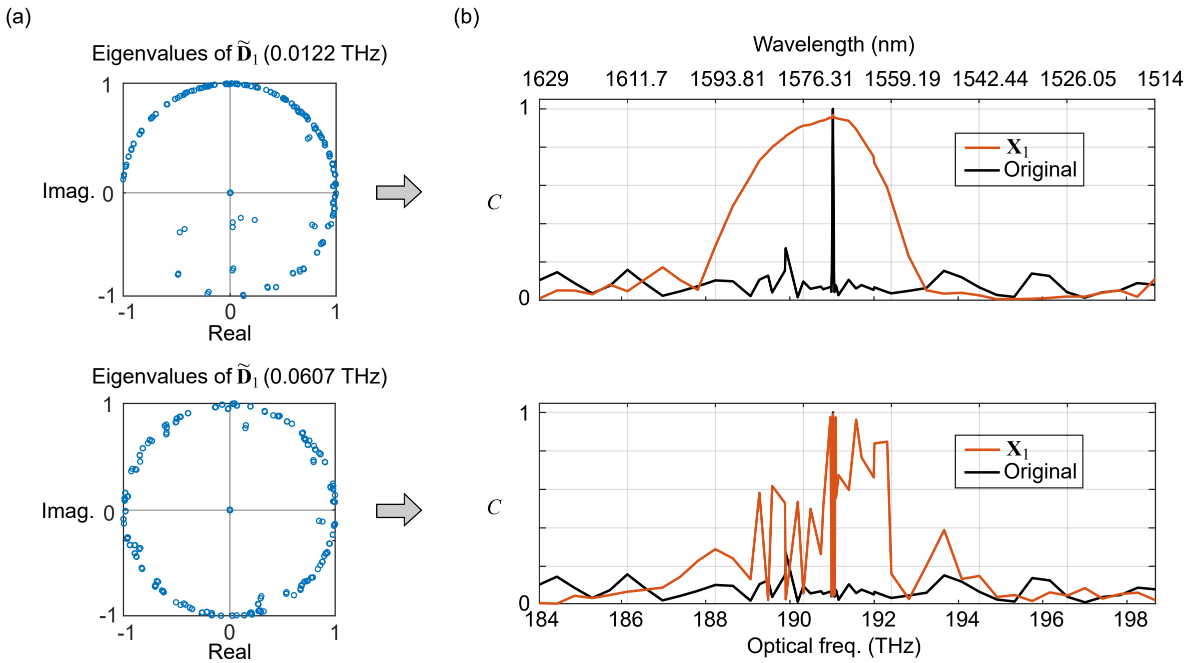

Figure S5: Evaluation of linear dispersion model with spectral sampling rate above (top) or below (bottom) the Nyquist criterion. (a) The eigenvalues of $\tilde{\mathbf{D}}(\delta\omega = 0.0122 \text{ THz})$ distribute across three fourth of the complex plane, and the eigenvalues of $\tilde{\mathbf{D}}(\delta\omega = 0.0607 \text{ THz})$ distribute across the entire complex plane. (b) The spectral correlation is continuous over ∼30 nm, while the spectral correlation is poor in the sub-Nyquist sampling condition due to inaccurate phase estimation.

For high order dispersion optimization, we need spectral sampling over a large spectrum to measure dispersion nonlinearity. However, similar to the linear dispersion, an insufficient sampling rate may also result in incorrect $\mathbf{X}_k, k \geq 2$ convergence. To show this, in a separate experiment with the same 1m-long 50-µm-core SI-MMF, we first computed the linear dispersion $\mathbf{D}_1$ centered at $\omega_0 = 191$ THz by using two msTMs of $(\omega_s, \delta\omega, \Omega) = (190.955, 0.015, 0.105)$ and $(190.6, 0.08, 0.8)$ THz. We then computed the eigenvalues of the linear dispersion $\mathbf{D}_1^{1c}(\delta\omega)$ of linear-dispersion-compensated $\mathbf{M}^{1c}(\omega) = \mathbf{D}_1^{-1}(\Delta\omega = \omega - \omega_0)\mathbf{M}(\omega)$ at two different spectral sampling $(\omega_s, \delta\omega, \Omega) = (189.134., 0.933, 3.732)$ and $(187.28, 1.86, 7.44)$ THz, respectively. As shown in Fig. S6(a), $\mathbf{D}_1^{1c}(\delta\omega = 0.933)$ has eigenvalues spread only half of the complex plane, and $\mathbf{D}_1^{1c}(\delta\omega = 1.86)$ almost covers the

entire plane. This informs that $\delta\omega = 0.933$ THz is a sufficient sampling rate for second order dispersion. Resuming back to regular higher order dispersion compensation, we then used the two msTMs with $\delta\omega = 0.933$ or 1.86 THz for optimizing second order dispersion of $\mathbf{M}(\omega)$ and tested the dispersion models. As plotted in Fig. S6(b), the spectral correlation with dispersion compensation to the second order is smooth over ~72 nm when using the msTM at $\delta\omega = 0.933$ THz. However, the spectral correlation is oscillating when using the msTM at $\delta\omega = 1.86$ Thz due to incorrect $\mathbf{X}_2$.

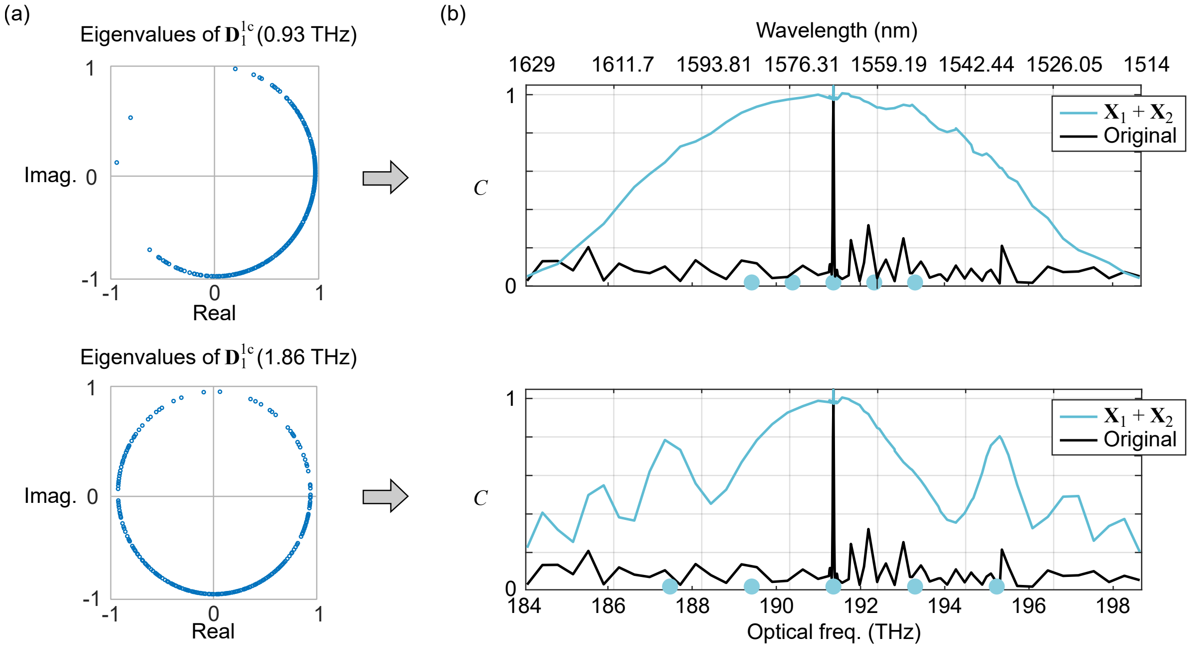

Figure S6: Evaluation of high order dispersion model with spectral sampling rate above (top) or below (bottom) the sampling criterion. (a) The eigenvalues of $\mathbf{D}_1^{1c}(\delta\omega = 0.933)$ distribute across half of the complex plane, and the eigenvalues of $\mathbf{D}_1^{1c}(\delta\omega = 1.86)$ distribute across the entire complex plane. (b) Accuracy of the constructed model using msTM of sampling step 0.933 (top) or 1.86 THz (bottom) for fitting the second order dispersion. The color dots label the corresponding spectral sampling points for high order dispersion optimization.

7 Generalization of dispersion model to various MMF

We verified the generalizability of our parametric dispersion model by testing it on different SI- and GI- MMF, as specified in Table S1. For each MMF, we constructed the corresponding dispersion model by using msTMs centered at 191 THz (1570 nm). We truncated the model to the kth order dispersion if no improvement was observed to the $k+1^{th}$ order. We then evaluated the dispersion model of order K with separately measured msTMs, and calculated the spectral correlation before and after dispersion compensation. The best attainable FWHM of computational spectral correlation bandwidth, $\delta\nu_e$, is recorded in Table S1. Therefore, we concluded that the model is valid for the various MMF regardless of refractive index profile, geometry, and length. Increased coupling, due to additional fiber perturbation or longer fiber length limits the spectral width of the model, although insufficiently dense spectral sampling for longer fibers due to experimental limitations may contribute to the observed performance.

With the corresponding constructed dispersion model of different MMF, we computed and studied the characteristics of their spectral-variant PMs. Here we show two representative results of the 1m-long few-mode fiber (FMF, indexed as "a" in Table S1) and the 1m-long GI-MMF (indexed as "h"). For the FMF, as shown in Fig. S7(a), we achieved a correlation above 0.8 over the full 115 nm spectrum after the dispersion compensation to $K = 3$, whereas the original correlation has multiple lobes and a coherence length of $\delta\nu = \sim 0.06$ THz. We identified 6 PMs in the FMF, with the corresponding permanence and mode profile shown in Fig. S7(b) and (c), and the PMs degenerate into H and V polarization. The group delays have a range of ~1.4 ps. For the GI-MMF, as shown

index	MMF spec.	refractive index	core size (um)	NA	length (m)	# of modes	radius of curvature (cm)	K	correlation BW (nm)
a	FG025LJA	SI	25	0.1	1	6	11	2	115
b	FG025LJA	SI	25	0.1	2	6	13	3	115
c	FG025LJA	SI	25	0.1	3	6	11	3	115
d	FG050LGA	SI	50	0.22	1	200	14	2	115
e	FG050LGA	SI	50	0.22	1	200	2.5	3	58
f	FG050LGA	SI	50	0.22	2	200	14	2	40
g	FG050LGA	SI	50	0.22	3	200	14	3	1.5
h	GIF625 (OM1)	GI	62.5	0.275	1	300	11	3	110
i	GIF625 (OM1)	GI	62.5	0.275	3	300	11	3	8
j	GIF50E (OM4)	GI	50	0.2	10	110	14	3	0

Table S1: Specifications and correlation bandwidths of various MMFs

in Fig. S8(a), the correlation with estimated linear dispersion (orange curve) has a pyramid shape, different to that in SI-MMFs. This is observed in other GI-MMFs and may be due to the parabolic refractive index profile and propagation properties. Nevertheless, we achieved 110 nm correlation bandwidth after the dispersion compensation to the third order. The permanence of each spectrally-dependent PM is shown in Fig. S8(b), where the average permanence is much reduced compared to the 1m-long 200-mode SI-MMF (indexed as "d") due to strong mode mixing and degeneracy within each mode group. We visualized the PM profiles in Fig. S8(c), which are scrambled speckle patterns instead of theoretical Hermite-Gaussian modes. The group delays are plotted in Fig. S8(d) and span across ∼5 ps. The results are not only consistent with theory and literature [6, 7], but the computational methods here avoid extensive experimental measurements.

We also describe here the computational spectral correlation in other MMF after dispersion compensation: The tightly coiled MMF (indexed as "e") has 7 loops with 2.5 cm radius of curvature and stronger modal scrambling compared to the MMF (indexed as "d") in the article. The spectral correlation after the first order dispersion compensation has a reduced bandwidth ∼2.5 THz (∼20 nm) compared to the same fiber in a loosely coiled condition. Nevertheless, the second order correction can further improve the correlation bandwidth to ∼58 nm. This indicates that the strong modal scrambling induces higher order dispersion; The 2-m-long MMF (indexed as "f"), compared to the identical MMF with 1 m length, has a shorter original $\delta\nu = \sim 20$ GHz (∼0.16 nm) and a reduced bandwidth of ∼18 nm after the first order dispersion compensation. This implies that the longer fiber length may result in more mode mixing. After correction to the second order, we can compensate additional dispersion and increase the correlation bandwidth to ∼40 nm; In the 3m-long OM1 GI-MMF (indexed as "i"), the modes within each group have similar propagation constants and strong mode mixing, and the MMF has an original coherence length of $\delta\nu = \sim 20$ GHz (∼0.16 nm). The mode mixing effect creates higher-order dispersion and compromises the reconstruction of dispersion model, where the longer fiber length exacerbates the effect. Nevertheless, with dispersion correction to the second order, we can improve the bandwidth to $\delta\nu_e = 8$ nm (∼1.5 THz); Finally, the 10-m-long OM4 MMF (indexed as "j") has a strong modal scrambling effect within individual mode groups. While the dispersion compensation to the first order slightly improves the correlation, the FWHM bandwidth remains the same. For the longer MMF "g," "i," and "j," where the first order model provides improvement to spectral correlation over a much shorter spectral range, the individual PMs computed at the center frequency similarly manifest fast correlation collapse over a small spectral range. This is in contradiction with previous reports of significant bandwidths in MMF in fibers as long as 100 m [7]. Also, the fact that the model fits well to shorter GI-MMF, where the mixing within individual mode groups is already in the fully coupled regime, suggests that our model, in principle, is able to describe dispersion in this regime. The observation that even the PMs only exhibit poor bandwidths in these longer fibers suggest that our measurement and phase stabilization strategy may be inadequate for the characterization of longer fibres with a larger pathlength offset.

8 Applicability of dispersion model to different measurement system

To test the generalization of our model to measurements from different hardware setups, we used a public msTM dataset of $(\omega_s, \delta\omega, \Omega) = (191.5, 0.0102, 5.22)$ THz ($N_\omega = 512$) acquired from loosely coiled OM1 MMF (graded-index, 62.5 µm core, 0.275 NA, 2 m fiber length, 420 modes) [8], and the TMs are represented in mode basis. We

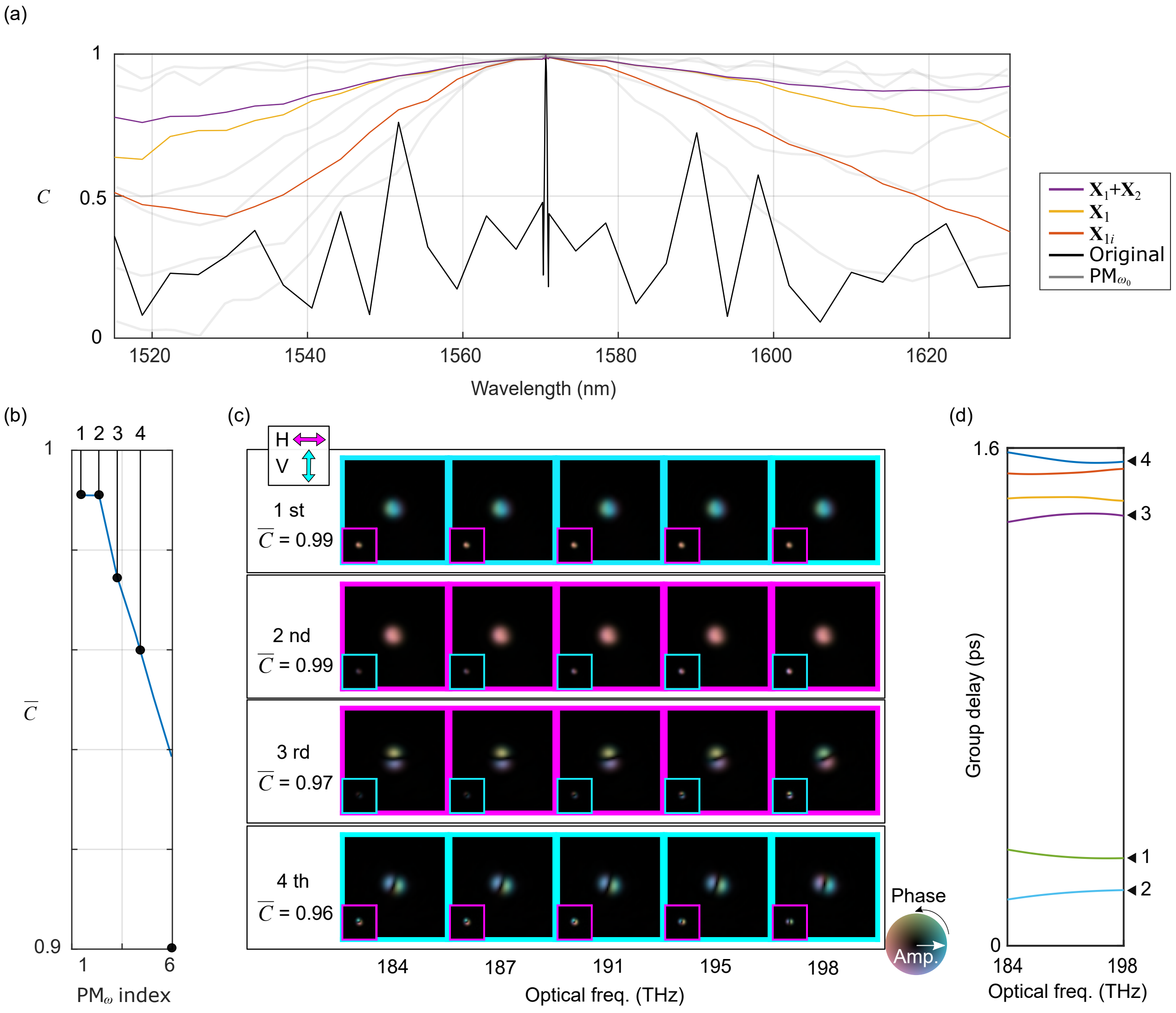

Figure S7: Computational spectral correlation in the 1m-long few-mode MMF ("a" in Table S1). (a) The spectral correlation before and after dispersion compensation to different orders: The orange curve is with initial linear dispersion estimation; The yellow curve is with first order dispersion optimized; The purple curve is with correction to the second order. The gray curves are the correlation of conventional PMs evaluated at the center frequency ω_0. (b) The permanence of the 6 spectral-variant PMs. (c) The complex profile of selected PMs across varying frequency (d) The group delays of individual PMs, with zoom-in on the selected PMs. The scale bar is 20 µm.

assumed the spatial channels are aligned and split the msTM into three subsets: $(\omega_s, \delta\omega, \Omega) = (194.07, 0.0102, 0.102)$ THz ($N_\omega = 11$) and $(\omega_s, \delta\omega, \Omega) = (191.53, 0.3068, 5.22)$ THz ($N_\omega = 18$) for fitting, and the rest of 483 TMs for testing. Figure S9 shows the spectral correlation before and after dispersion compensation to different orders. The model to the third order captures dispersion across the total 41 nm spectrum with excellent accuracy. As a result, the architecture is robust and can be applied to different MMF calibration system with different TM representations.

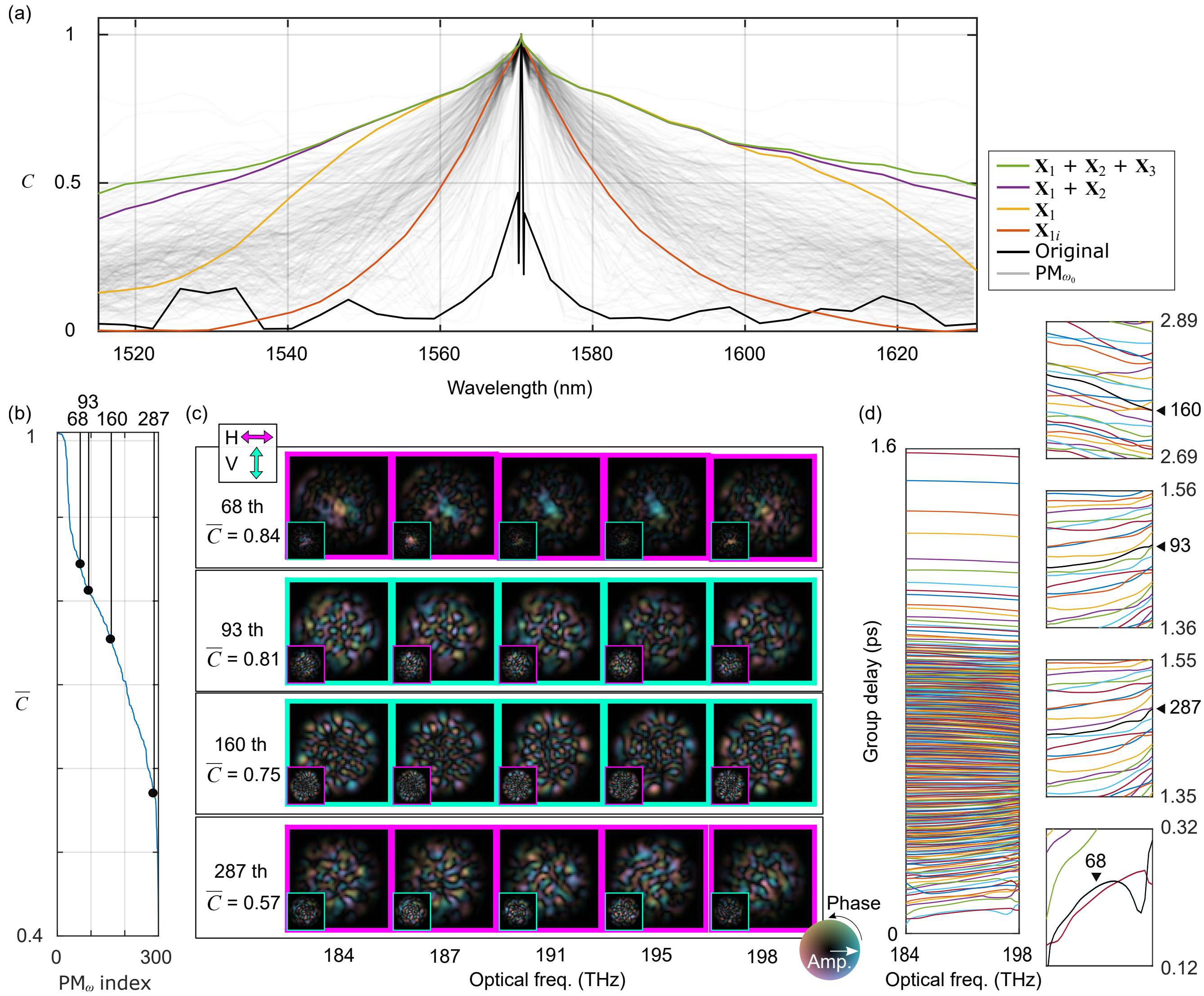

Figure S8: Computational spectral correlation in the 1m-long GI-MMF (”h” in Table S1). (a) The spectral correlation before and after dispersion compensation to different orders: The orange curve is with initial linear dispersion estimation; The yellow curve is with first order dispersion optimized; The purple curve is with correction to the second order; The green curve is with correction to the third order. The gray curves are the correlation of conventional PMs evaluated at the center frequency ω_0. (b) The permanence of the 300 spectral-variant PMs. (c) The complex profile of selected PMs across varying frequency (d) The group delays of individual PMs, with zoom-in on the selected PMs. The scale bar is 20 µm.

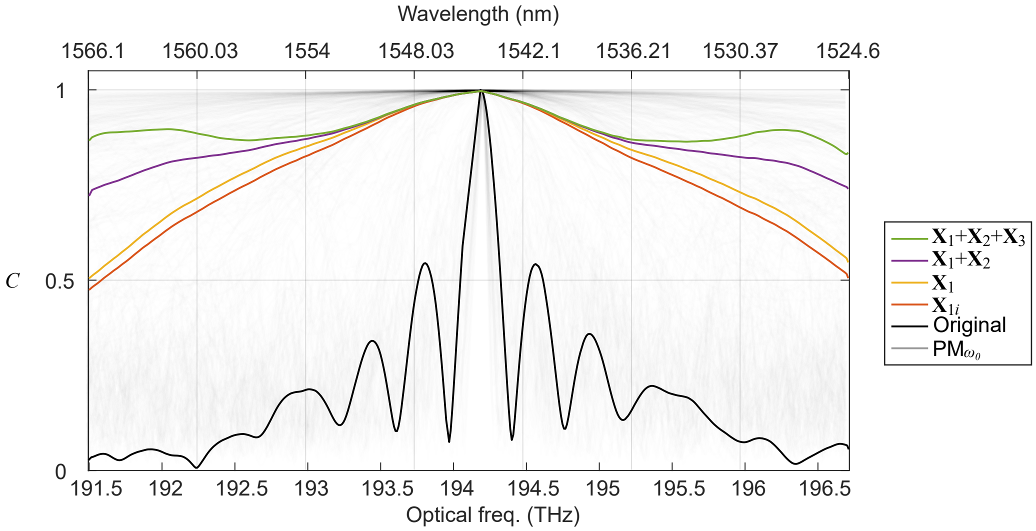

Figure S9: Computational spectral correlation in 2m-long GI-MMF using the public dataset.

References

[1] Etienne Cuche, Pierre Marquet, and Christian Depeursinge. Spatial filtering for zero-order and twin-image elimination in digital off-axis holography. *Appl. Opt.*, 39(23):4070–4075, Aug 2000.

[2] Wen Xiong, Philipp Ambichl, Yaron Bromberg, Brandon Redding, Stefan Rotter, and Hui Cao. Principal modes in multimode fibers: exploring the crossover from weak to strong mode coupling. *Optics Express*, 25(3):2709, 2017.

[3] Mario Lezcano-Casado and David Martínez-Rubio. Cheap orthogonal constraints in neural networks: A simple parametrization of the orthogonal and unitary group, 2019.

[4] Awad H. Al-Mohy and Nicholas J. Higham. A new scaling and squaring algorithm for the matrix exponential. *SIAM Journal on Matrix Analysis and Applications*, 31(3):970–989, 2009.

[5] Larry Armijo. Minimization of functions having Lipschitz continuous first partial derivatives. *Pacific Journal of Mathematics*, 16(1):1 – 3, 1966.

[6] Mahdieh B. Shemirani, Wei Mao, Rahul Alex Panicker, and JosephM. Kahn. Principal modes in graded-index multimode fiber in presence of spatial- and polarization-mode coupling. *J. Lightwave Technol.*, 27(10):1248–1261, May 2009.

[7] Joel Carpenter, Benjamin J. Eggleton, and Jochen Schröder. Observation of Eisenbud-Wigner-Smith states as principal modes in multimode fibre. *Nature Photonics*, 9(11):751–757, 2015.

[8] Joel Carpenter, Benjamin J. Eggleton, and Jochen Schröder. Comparison of principal modes and spatial eigenmodes in multimode optical fibre. *Laser & Photonics Reviews*, 11(1):1600259, 2017.
